# CRISPR‐Cas9‐Loaded Theranostic Liposomes for Enhancing Radiosensitization of Prostate Cancer through POLD4 Gene Editing under Real‐Time MRI Monitoring

**DOI:** 10.1002/advs.202519704

**Published:** 2026-01-07

**Authors:** Xuhui Fan, Ruru Zhang, Linjun Yang, Shixiong Chen, Meijuan He, Yongqiang Wang, Linjie Huang, Jianfeng Zeng, Shuwang Wu, Mingyuan Gao, Han Wang

**Affiliations:** ^1^ Department of Radiology Shanghai General Hospital, Shanghai Jiao Tong University School of Medicine Shanghai P. R. China; ^2^ State Key Laboratory of Radiation Medicine and Protection School for Radiological and Interdisciplinary Sciences (RAD‐X) Collaborative Innovation Center of Radiation Medicine of Jiangsu Higher Education Institutions Soochow University Suzhou P. R. China; ^3^ Department of Bone and Joint Surgery Renji Hospital Shanghai Jiao Tong University School of Medicine Shanghai P. R. China; ^4^ School of Life Sciences Suzhou Medical College Soochow University Suzhou P. R. China; ^5^ Shanghai General Hospital Branch of National Center for Translational Medicine (Shanghai) Shanghai P. R. China; ^6^ Jiading Branch of Shanghai General Hospital Shanghai P. R. China

**Keywords:** DNA repair, lipid nanoparticles, magnetic resonance imaging, prostate cancer, radiosensitization

## Abstract

Radiotherapy is a fundamental treatment for prostate cancer; however, its therapeutic efficacy is frequently limited by radioresistance mediated through DNA repair mechanisms and other biological factors. Although gene therapy holds promise for overcoming such resistance, identifying effective radiosensitization targets and developing efficient gene delivery systems remain practically challenging. In this study, transcriptomic analysis of radiotherapy‐treated prostate cancer cells revealed a marked upregulation of DNA polymerase delta subunit 4 (POLD4), a target that has not been systematically studied. To evaluate the potential of POLD4 for overcoming radioresistance, CRISPR‐Cas9‐based plasmids along with ultrasmall superparamagnetic iron oxide nanoparticles (USPIONs) were encapsulated within cationic liposomes for achieving an MRI‐trackable gene delivery platform (plasmid and iron oxide co‐loaded liposomes, termed PIO@Lipo). Comprehensive in vitro and in vivo studies demonstrated that PIO@Lipo enabled efficient POLD4 knockdown. Furthermore, PIO@Lipo synergized with radiotherapy to induce extensive DNA damage, promote tumor cell apoptosis, and remodel the immunosuppressive microenvironment. Notably, PIO@Lipo displayed superior MRI contrast enhancement capability and passive tumor targeting ability. In conclusion, this study has identified POLD4 as a potent target for radiosensitization, capable of disrupting DNA damage‐repair homeostasis through MRI‐monitored gene editing, thereby offering a promising strategy to enhance the efficacy of radiotherapy in prostate cancer.

## Graphical abstract

This study identifies POLD4 as a potential prostate cancer radiosensitization target through transcriptome sequencing. By encapsulating POLD4‐targeted CRISPR/Cas9 plasmids and USPIONs in cationic liposomes, an MRI‐monitored gene‐editing platform is established. Relevant experiments demonstrate that knocking down POLD4 expression disrupts the balance between tumor DNA damage and repair, thereby eliciting potent radiosensitization.

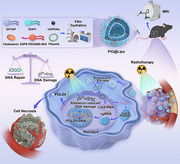

## 1 Introduction

Prostate cancer is one of the most commonly diagnosed malignancies and the second leading cause of cancer‐related mortality among men worldwide [1]. Radiotherapy remains one of the fundamental treatment methods for locally progressed prostate cancer, offering significant clinical benefits to intermediate‐ and high‐risk patients [2]. However, the development of radioresistance in prostate cancer presents a major clinical challenge, often resulting in treatment failure and disease progression [3]. The radioresistance occurs through multiple mechanisms: the activation of DNA damage repair pathways (e.g., PARP‐mediated repair systems), which effectively correct radiation‐induced DNA lesions [4]; intratumoral heterogeneity, which allows radiation‐resistant subclones to survive and proliferate under therapeutic pressure [5]; and the hypoxia‐mediated resistance, which induces metabolic reprogramming toward radioresistant phenotypes [6]. These mechanisms act synergistically to reduce the effectiveness of radiotherapy, frequently necessitating dose escalation, thereby increasing the risk of damage to normal tissues without guaranteeing long‐term tumor control [3]. Therefore, the development of effective targeted therapeutic strategies to counteract these resistance mechanisms, particularly through the inhibition of DNA damage repair pathways, is crucial for overcoming radioresistance and improving the efficacy of radiotherapy.

Gene therapy holds significant promise for overcoming radioresistance through the molecular “reprogramming” of tumor cells [7]. Anai et al. observed that combining phosphatase and tensin homolog (PTEN) gene overexpression with radiotherapy significantly suppressed tumor growth compared to radiotherapy alone, attributable to the inhibition of angiogenesis and suppression of cellular proliferation [8]. However, gene therapy for overcoming radioresistance continues to face several critical challenges. First, current molecular targets, e.g., PTEN, are insufficient to counteract subsequent tumor DNA repair mechanisms, limiting optimal radiosensitization [9, 10]. Second, viral vectors pose risks of immune activation and genomic instability, whereas non‐viral delivery systems, including nanoparticle‐based carriers, exhibit limited tumor accumulation and inconsistent gene silencing efficiency [11, 12, 13, 14]. Third, the lack of real‐time monitoring of gene delivery impedes the optimization of therapeutic agents and targeting accuracy [15]. Therefore, identifying novel radiosensitizing gene targets and developing trackable gene delivery platforms remain essential for enhancing therapeutic outcomes.

Among various non‐viral gene delivery systems, cationic liposomes have proven highly effective for gene delivery [16]. Their positive surface charge facilitates efficient complexation with negatively charged nucleic acids and therapeutic agents through electrostatic interactions [17, 18]. Due to their favorable cellular uptake and robust endosomal escape properties, liposomes provide high gene drug loading capacity and enhanced gene therapeutic efficacy in gene therapy [18, 19, 20, 21]. Compared to conventional approaches, this method presents several advantages, including improved biocompatibility with minimal immune activation, the ability to co‐deliver gene‐editing components and tracking agents, and enhanced genome editing efficiency. As a widely utilized gene‐editing tool in eukaryotic systems, the Clustered Regularly Interspaced Short Palindromic Repeats (CRISPR)/CRISPR‐associated protein 9 (Cas9) system functions as a programmable molecular scissors system, employing a guide RNA (gRNA) to direct the Cas9 nuclease to specific DNA sequences, where it induces double‐strand breaks enables precise genome modifications [22]. Common CRISPR delivery methods include ribonucleoprotein (RNP) and plasmid‐based approaches. The RNP approach involves direct assembly of Cas9 protein and gRNA, enabling efficient editing of target genes within a short timeframe [23]. However, it suffers from laborious Cas9 protein synthesis and instability, primarily due to proteolytic hydrolysis. Plasmids, on the other hand, are double‐stranded circular DNA constructs that exhibit enhanced stability, can be efficiently encapsulated within liposomes, and enable scalable production via bacterial amplification. Their main limitation is the potential off‐target risks associated with prolonged expression, which necessitates rigorous safety testing to ensure applicability [24]. In a recent study, Yang et al. successfully employed liposomes for delivering CRISPR‐Cas9 plasmids, demonstrating that liposome‐encapsulated Cas9 plasmid could achieve reduce the expression of the target gene by 52.3% through tissue‐specific genome editing [25]. Therefore, investigating radioresistance‐related signaling pathways, identifying effective radiosensitizing targets, and delivering liposomes‐encapsulated genetic therapeutics represent a promising strategy to overcome tumor radioresistance.

**FIGURE 1:**
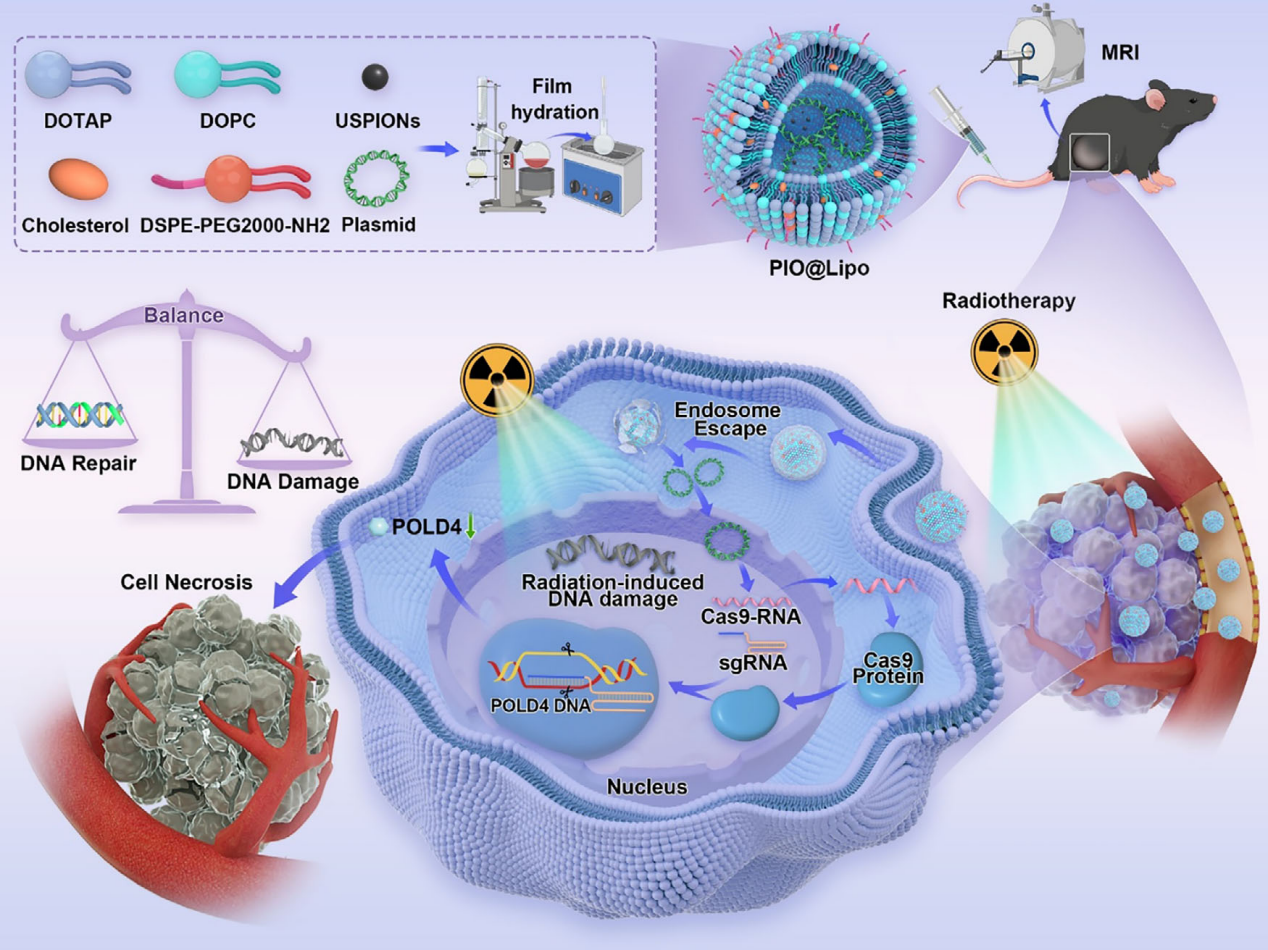
Schematic workflow for the preparation process and in vivo molecular mechanisms of the MRI‐trackable gene delivery platform.

In this study, POLD4 was selected as a critical gene target for radiosensitization in prostate cancer based on transcriptome sequencing analysis. To investigate the role of POLD4 in modulating radiation response, a CRISPR/Cas9 plasmid specifically targeting POLD4 was constructed and co‐encapsulated with USPIONs into cationic liposomes (termed PIO@Lipo). Experimental results demonstrated that the resulting PIO@Lipo, which is detectable by magnetic resonance imaging (MRI), effectively induced POLD4 gene silencing and thereby enhanced tumor sensitivity to radiation therapy. The overall experimental workflow is illustrated in Figure 1.

## 2 Results and Discussion

### 2.1 Exploring New Radiosensitization Gene Target

**FIGURE 2:**
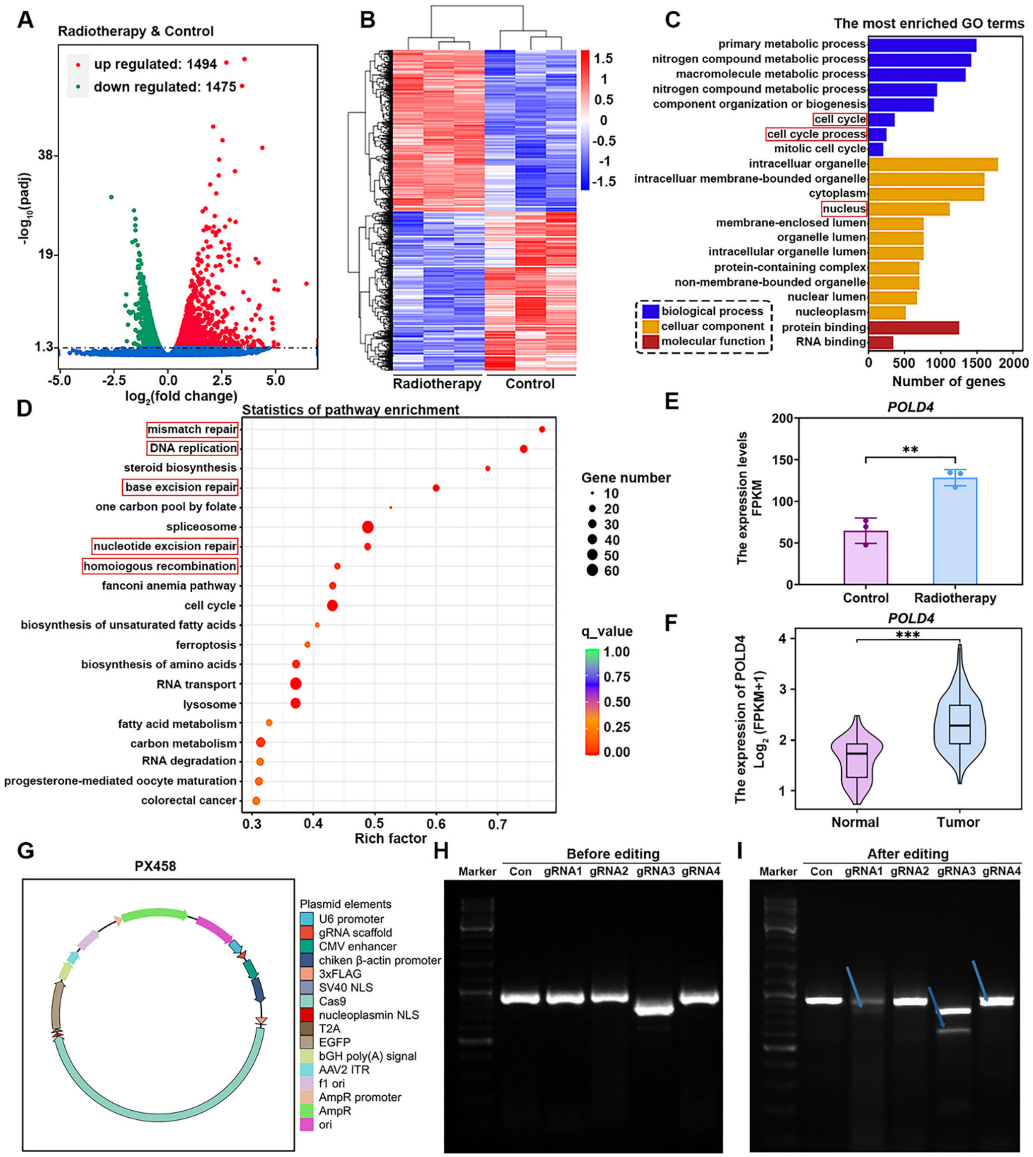
Sequencing analysis of prostate cancer cells and POLD4‐targeted CRISPR/Cas9 plasmid development. A) Volcano plot of sequencing results, showing DEGs following radiotherapy (red: upregulated; green: downregulated), with an adjusted *p*‐value < 0.05 indicating statistically significant. B) Heatmap comparing the expression patterns of DEGs between the radiotherapy and control groups, showing significant intergroup variations. C, D) GO and KEGG pathway analyses, with red boxes highlighting the gene functions and pathways of particular interest in this study. E) Comparative analysis of POLD4 mRNA expression levels between the control and radiotherapy groups (3v3) based on sequencing results. F) Comparison of POLD4 expression levels between normal and tumor tissues from the TCGA‐PRAD cohort. G) Schematic diagram of the CRISPR/Cas9 PX458 plasmid structure, with key components labeled on the right side. H, I) H panel shows the agarose gel electrophoresis results of the PCR‐amplified POLD4 gene, while Panel I displays the DNA fragments generated by T7E1 digestion at the targeted editing site, with the expected band indicated by the arrow. Data from independent experiments were expressed as mean ± SD, with significance levels denoted as ^*^ *p* < 0.05, ^**^ *p* < 0.01, ^***^ *p* < 0.001, and ^****^ *p* < 0.0001. Consistently, all subsequent figures herein use the same data presentation and significance notation.

This study first investigated 6 Gy radiation‐induced transcriptional alterations in RM‐1 prostate cancer cells, and 2,969 differentially expressed genes (DEGs) were identified with adjusted *p*‐value < 0.05 in comparison to the control group (Figure 2A). As anticipated, hierarchical clustering analysis demonstrated distinct expression patterns of these genes after irradiation (Figure 2B). Further analysis on these differentially expressed genes through gene ontology (GO) enrichment revealed that these DEGs were predominantly enriched in biological processes associated with cell cycle regulation and cellular components localized to the nucleus (Figure 2C). Moreover, Kyoto encyclopedia of genes and genomes (KEGG) pathway analysis showed a significant enrichment of DEGs in key DNA repair pathways, including mismatch repair, DNA replication, base excision repair, nucleotide excision repair, homologous recombination repair, etc (Figure 2D). These findings indicate that prostate cancer cells activate a comprehensive network of DNA damage repair mechanisms in response to radiation exposure. Notably, POLD4 was identified as a common regulatory component across these pathways, with significantly upregulated expression levels as shown in Figure 2E. POLD4 is an essential subunit of the DNA polymerase delta complex and plays key roles in DNA replication and repair processes [26]. When the DNA polymerase encounters a damaged template strand, POLD4 promotes replication fork reversal to facilitate lagging‐strand synthesis, thereby maintaining genomic integrity [27]. Bioinformatic analysis on TCGA‐PRAD transcriptomic data further revealed a significantly elevated POLD4 expression in prostate tumor tissues compared to adjacent normal counterparts (Figure 2F). This finding is consistent with the study by Jiang et al., who reported that POLD4 was frequently upregulated in various malignancies and significantly associated with poor patient survival, suggesting its potential not only as a prognostic biomarker but also as a promising therapeutic target [28]. Previous studies have demonstrated that POLD4 knockout significantly enhanced lymphoma cell sensitivity to camptothecin, a chemotherapeutic agent [27], while Gu et al. found that it didn't affect the viability of normal cells, but reduced liver tumor incidence [29]. Despite being an essential component of DNA damage repair machinery, the radiosensitization role of POLD4 has not received systematic investigations. Based on the above findings and evidence, it is reasonable to hypothesize that knockout of POLD4 may enhance prostate cancer radiosensitivity without compromising normal tissue function.

To verify this hypothesis, five CRISPR‐Cas9 plasmids were designed, and each plasmid contains a different single‐guide RNA (sgRNA) for targeting POLD4 in prostate cancer cells. The plasmid structures and corresponding sgRNA targeting sequences are shown in Figure 2G and Table S1. Each plasmid primarily consists of a Cas9 expression cassette, a target‐specific sgRNA sequence for CRISPR‐mediated genome editing, and an enhanced green fluorescent protein (EGFP) marker for monitoring transfection efficiency. After 48‐h plasmid transfection, cellular DNA was extracted and amplified via polymerase chain reaction (PCR) using primers listed in Table S2, resulting in target DNA fragments as shown in Figure 2H. The T7 endonuclease I (T7E1) assay was subsequently conducted to assess the efficiency of plasmid transfection by detecting the cleavage of target DNA. The results revealed that sgRNA3 produced the optimal results, exhibiting clear cleavage bands different from the original intact DNA (Figure 2I). Accordingly, the plasmid containing sgRNA3 was selected for the subsequent experiments.

### 2.2 Characterization of Liposomes Co‐Loaded with Plasmid and USPIONs

**FIGURE 3:**
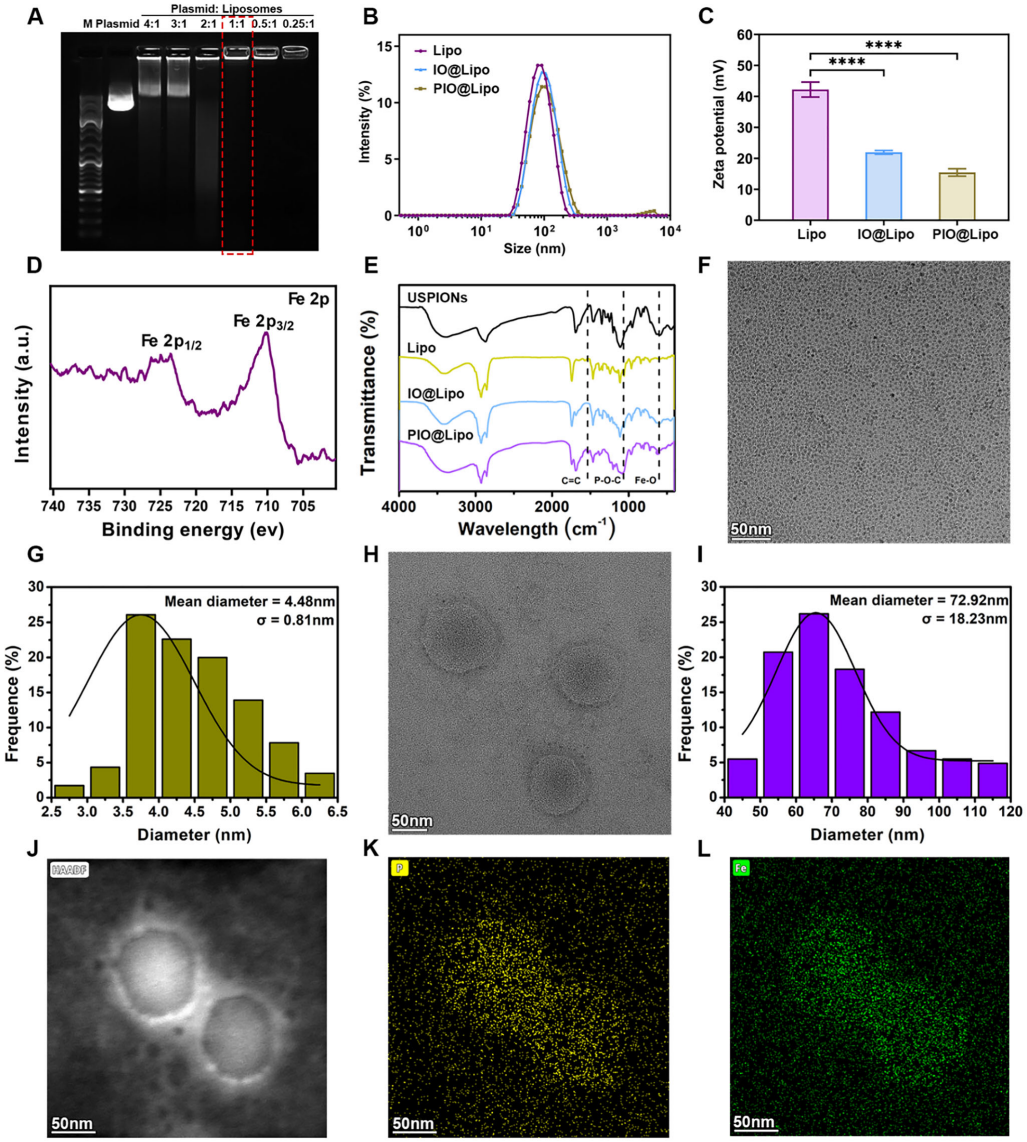
Characterization of the nanoparticles utilized in this study. A) Agarose gel retardation assay of plasmid and cationic liposome complexes at various mass ratios, where the absence of free plasmid migration outside the loading wells demonstrates effective plasmid encapsulation. B, C) Dynamic Light Scattering (DLS) results: Panel B shows the hydrodynamic diameter of liposomes, and Panel C presents the surface zeta potential of liposomes. D) XPS spectra of PIO@Lipo showing characteristic peaks corresponding to the Fe 2p orbitals of USPIONs. E) FTIR spectra of nanoparticles with dashed lines indicating the characteristic vibrational bands of functional groups. F–I) TEM images of USPIONs and PIO@Lipo with corresponding particle size distribution statistics. J–L) TEM elemental mapping of PIO@Lipo: HAADF‐STEM image (J), P element distribution (K), and Fe element distribution (L).

The agarose gel retardation assay was employed to determine the mass ratio of plasmid to liposomes. The results demonstrated complete plasmid encapsulation at a 1:1 mass ratio, which confirms the liposomes’ superior ability to encapsulate plasmids (Figure 3A). Quantitative analysis using the PicoGreen assay quantified plasmid DNA concentrations both outside and inside the liposomes, yielding an encapsulation efficiency (EE%) of 91.8%. The hydrodynamic diameter of the resulting liposomes ranges from 72.1 to 94.8 nm, with a polydispersity index (PDI) of 0.20–0.25 after co‐loading with plasmid and USPIONs nanoparticles (Figure 3B). The empty liposomes (Lipo) exhibit a zeta potential of 42.2 mV. After loading with USPIONs (IO@Lipo), the zeta potential was decreased to 21.9 mV. After being incorporated with the plasmid (PIO@Lipo), the zeta potential further dropped to 15.4 mV (Figure 3C). USPIONs were modified with bisphosphonate‐PEG, endowing their surface with negative charges from the bisphosphonate groups. Thus, the encapsulation mechanism of USPIONs is consistent with that of plasmids, both relying on electrostatic interaction. X‐ray photoelectron spectroscopy (XPS) analysis on PIO@Lipo revealed the characteristic Fe_3_O_4_ peaks, confirming successful incorporation of USPIONs (Figure 3D). Fourier transform infrared spectroscopy (FTIR) results (Figure 3E) demonstrated the characteristic peaks corresponding to the unsaturated C═C vibrational bands of liposomes, P─O─C band of plasmids, and Fe─O band of USPIONs, thereby confirming the successful incorporation of both plasmids and USPIONs within the liposomes. Figure 3F,G display transmission electron microscopy (TEM) images and size distribution profile of the USPIONs, showing an average diameter of 4.5 ± 0.8 nm. Meanwhile, Figure 3H,I display a membrane‐structured morphology of PIO@Lipo, which had an average diameter of 72.9 nm. The optimized size of PIO@Lipo falls within the ideal range for the enhanced permeability and retention (EPR) effect to enable effective tumor uptake [30]. It is well accepted that particles larger than 200 nm may lead to pulmonary embolism due to mechanical entrapment in the capillary network and are rapidly cleared by the reticuloendothelial system (RES), predominantly accumulated in the liver and spleen [31], while particles smaller than 20 nm are subject to rapid renal clearance and exhibit reduced efficiency in encapsulating plasmid [32]. Notably, the liposomes used in this study consist of DOTAP, DOPC, cholesterol, and DSPE‐PEG‐NH_2_ at a molar ratio of 50:10:37:3, which is consistent with the proportions reported in some previous studies [33, 34, 35]. Typically, the composition of such liposomes is 50:10:38.5:1.5 [33]. However, numerous recent studies have suggested that an appropriate increase in the proportion of PEGylated lipids can enhance encapsulation efficiency, in vivo stability of liposomes, and antifouling ability, while reducing uptake by the RES [36, 37, 38]. Considering the applicability of in vivo transfection, the proportion of PEGylated lipids was adjusted to 3% in this study. Figure 3J–L present the TEM elemental mapping of PIO@Lipo, demonstrating a high enrichment of phosphorus (P) derived from plasmids and partially from liposomes, as well as iron (Fe) originating from USPIONs within the interior of the liposomes.

### 2.3 PIO@Lipo Enhance the Efficacy of Radiotherapy by Suppressing Tumor Growth and Inducing Apoptosis

**FIGURE 4:**
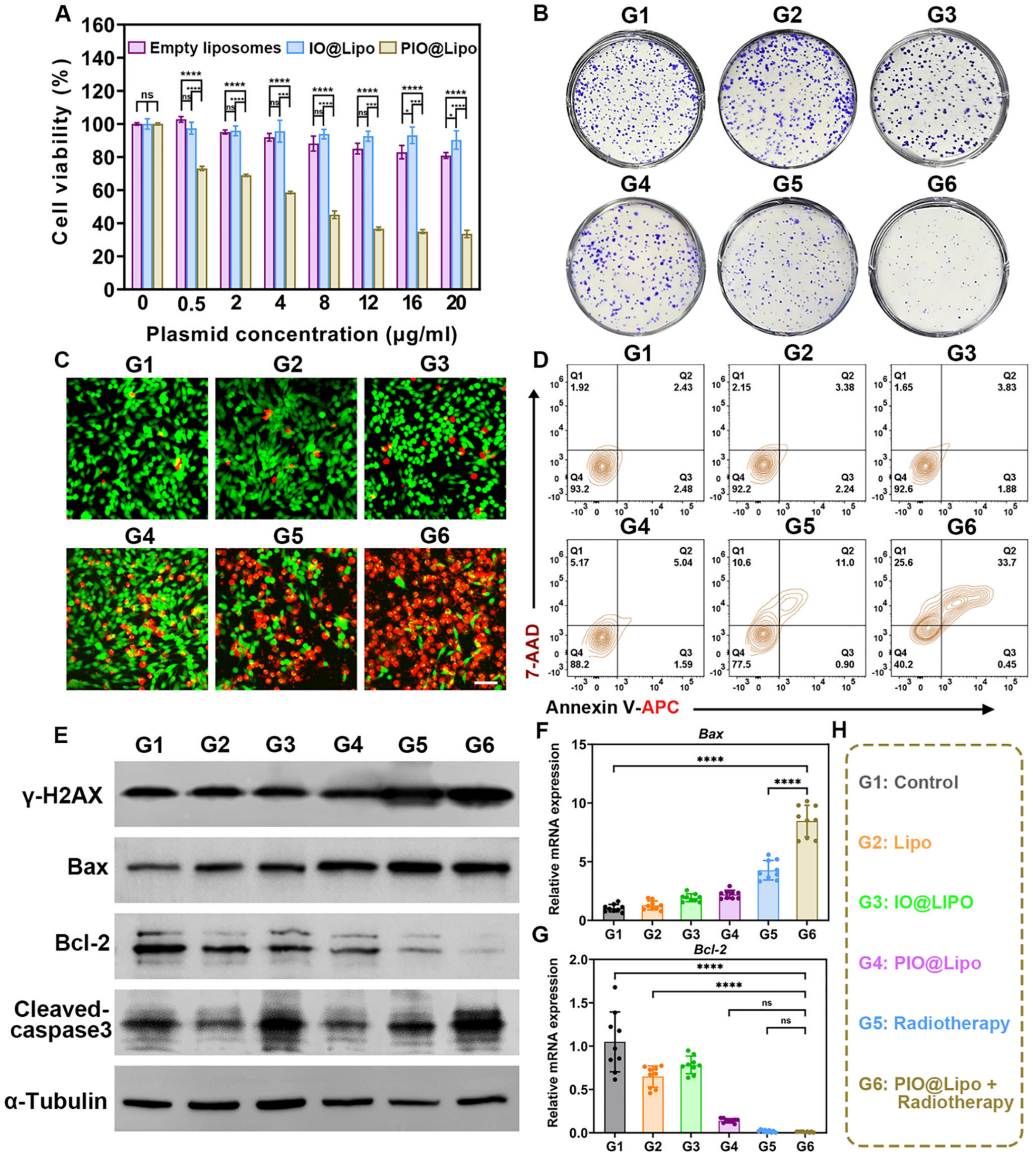
G1 represents the control group, G2 the empty liposome group (Lipo), G3 the USPION‐loaded liposome group (IO@Lipo), G4 the plasmid and USPION co‐loaded liposome group (PIO@Lipo), G5 the radiotherapy group, and G6 the PIO@Lipo combined with radiotherapy group. A) CCK‐8 assay results showing the impact of nanoparticles on prostate cancer cell viability. B) Colony formation assay demonstrating the effects of various liposome formulations and radiotherapy on prostate cancer cells. C) Live/dead staining assay, with a white scale bar of 50 µm included for reference. D) Flow cytometry apoptosis assay of each group, with cell distribution presented as contour plots. E) Western blot analysis assessing the expression of DNA damage‐ and apoptosis‐related markers in prostate cancer cells after treatment. F, G) qPCR results of the expression level of Bax and Bcl‐2. H) The specific therapeutic interventions administered to G1‐G6.

In this study, G1‐G6 were used to represent different experimental groups: G1 as the control group, G2 as the empty liposome group (Lipo), G3 as the USPION‐loaded liposome group (IO@Lipo), G4 as the plasmid and USPIONs co‐loaded liposome group (PIO@Lipo), G5 as the radiotherapy group, and G6 as the PIO@Lipo combined with radiotherapy group. The cell counting kit‐8 (CCK‐8) assay was employed to investigate the effects of Lipo, IO@Lipo, and PIO@Lipo on tumor cell proliferation (Figure 4A). The results demonstrated that neither Lipo nor IO@Lipo exerted a significant impact on tumor cell viability. In contrast, the introduction of plasmid led to a noticeable reduction in tumor cell viability at a concentration of 0.5 µg/mL, with cell viability being further decreased to 45% at 12 µg/mL. As shown in Figure 4B and Figure S1, the colony formation assay revealed no significant difference between the control and Lipo groups, whereas IO@Lipo reduced the average colony count from 375 to 284 (*p* < 0.0001). PIO@Lipo treatment resulted in a further reduction in colony number, although less pronounced than that observed with radiotherapy alone. Notably, the combination of radiotherapy and PIO@Lipo exhibited synergistic effects by decreasing colony number to 81. Both CCK‐8 and colony formation assays demonstrated that the combination of PIO@Lipo with radiotherapy synergistically inhibited the proliferation of prostate cancer cells. Furthermore, live/dead staining (Figure 4C) revealed that apoptotic cells (red fluorescence) were minimal in groups G1‐G3, whereas a significant increase in apoptosis was observed in groups G4‐G6. Remarkably, nearly all cells in G6 exhibited red fluorescence, with minimal green fluorescence (indicating live cells). Flow cytometry analysis of apoptosis (Figure 4D) quantitatively corroborated these observations, revealing that the percentage of late apoptotic cells remained low (∼3%) in G1‐G3, but was increased to 11.0% in the radiotherapy group (G5), and further to 33.7% in the combined radiotherapy/PIO@Lipo group (G6), clearly demonstrating the strong synergistic antitumor effects of this radiation/gene combined therapy. After lysis of the cells and extraction of total proteins from the aforementioned cells, western blot analysis was carried out, and a marked upregulation of the DNA damage marker γ‐H2AX was observed in both G5 and G6 after radiotherapy, with G6 showing higher expression than G5. This was accompanied by increased levels of pro‐apoptotic proteins Bax and cleaved‐caspase3, as well as decreased expression of anti‐apoptotic Bcl‐2, clearly indicating the robust activation of the mitochondrial (intrinsic) apoptosis pathway, ultimately leading to programmed cell death in tumor cells (Figure 4E). The quantitative PCR (qPCR) results (Figure 4F,G) further confirmed the mRNA‐level changes in Bax and Bcl‐2 within this apoptotic pathway. Figure 4H illustrates the detailed grouping information for the aforementioned cohorts G1‐G6. These results demonstrated that the combination of radiotherapy and gene therapy of G6 exhibited the most potent antitumor efficacy, significantly surpassing the groups receiving gene therapy alone (G4) or radiotherapy alone (G5). This synergistic effect is attributed to the gene therapy's capacity to compromise the genomic repair mechanisms of tumor cells receiving radiation, thereby enhancing radiosensitivity [39].

To provide additional evidence for the radiosensitizing effect of POLD4, this study further included sgRNA1 (sgRNA3 used in previous experiments) and designed an sgRNA‐resistant POLD4 cDNA plasmid (Figure S2). This plasmid was engineered by introducing synonymous mutations into the sgRNA target site, thereby restoring the ability of cells to express POLD4. Figure S3 confirms that both sgRNA1 and sgRNA3 effectively knock down POLD4 protein expression and both increase γ‐H2AX expression following radiotherapy. In addition, treatment with the sgRNA‐resistant cDNA plasmid restores POLD4 expression levels and reduces γ‐H2AX expression. Furthermore, this study demonstrated through the same phenotypic assays (including colony formation assay, flow cytometric analysis of apoptosis, and live/dead cell staining assay) that POLD4 knockdown induced by both sgRNAs effectively elicited a radiosensitizing effect (Figures S4–S6). Restoration of POLD4 expression attenuated this radiosensitizing effect, which further confirmed that POLD4 is indeed a valid target for radiosensitization. Current literatures indicate that POLD4 participates in DNA damage repair pathways. However, its specific mechanisms and therapeutic implications in enhancing radiotherapy efficacy remain not fully elucidated. POLD4, a subunit of the DNA polymerase delta (Polδ) complex, plays a key role in break‐induced replication (BIR)‐mediated DNA repair. Depletion of POLD4 reduces BIR efficiency, particularly during the G2/M phase of the cell cycle, resulting in increased genomic instability in tumor cells [40]. Given that the G2/M phase is also the most radiosensitive phase due to chromatin condensation and diminished DNA repair capacity, POLD4 deficiency synergizes with radiotherapy by further destabilizing tumor genomes and promoting apoptosis. Together, these effects establish a synthetic lethality between POLD4 deficiency and radiotherapy, offering a promising therapeutic strategy for radiosensitization.

### 2.4 Transfection Efficiency of PIO@Lipo and Its Impact on Gene Editing Pathways

**FIGURE 5:**
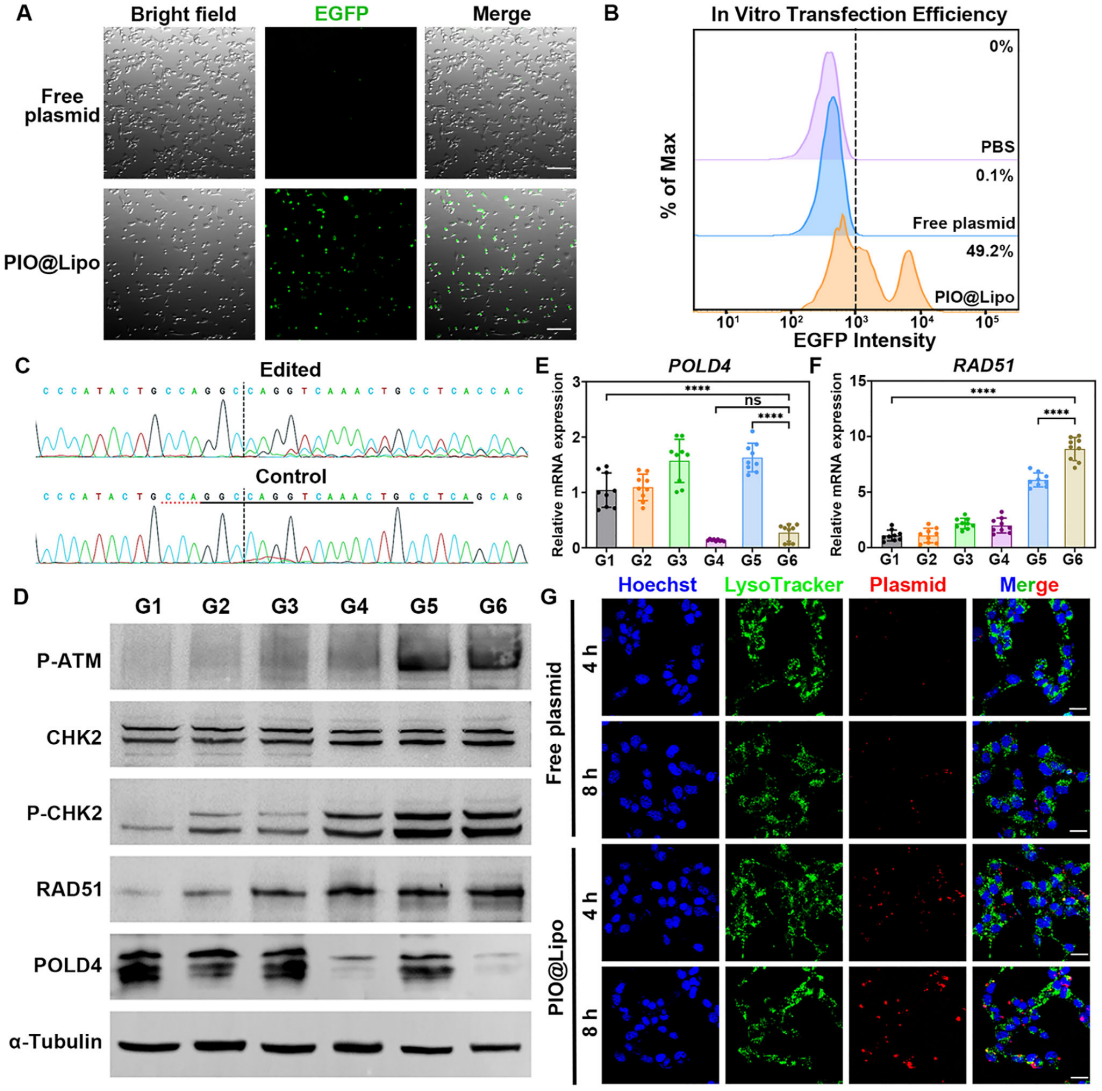
(A, B) Confocal microscopy and flow cytometry analysis of EGFP expression in cells (scale bar: 50 µm). C) Sanger sequencing results of PIO@Lipo‐edited cells showing CRISPR/Cas9 cleavage sites (dashed lines) and subsequent sequence variations compared to the control group. D) Western blot analysis on POLD4 expression and DNA damage repair pathway after treatment. E, F) qPCR results on POLD4 and RAD51 gene expression levels. G) Lysosomal escape assay of iFluor 647‐labeled free plasmids and PIO@Lipo (scale bar: 30 µm).

First, we evaluated the transfection efficiency of PIO@Lipo using the EGFP reporter element carried by the plasmids. Figure 5A presents confocal microscopy images of the transfection results, compared with free plasmids. PIO@Lipo significantly enhanced plasmid transfection efficiency, leading to a considerable proportion of cells exhibiting EGFP fluorescence. Flow cytometric quantification (Figure 5B) demonstrated that the proportion of successfully transfected cells reached 49.2%, confirming the superior transfection capability of PIO@Lipo. Additionally, flow cytometric quantification of in vivo tumor tissue cells indicated that the proportion of transfected cells reached 14.9% (Figure S7). Figure 5C presents the Sanger sequencing results of the targeted editing sites following POLD4 gene modification mediated by PIO@Lipo. In the control group without CRISPR/Cas9 editing, the POLD4 sequence remains intact. However, after gene editing, the genomic sequence in the downstream of the dashed line shows significant disruption. Figure S8 provides a more direct visualization of the differences between the two groups. In the downstream of the dashed line, the edited sample exhibits pronounced genomic discrepancies compared to the control. Additionally, in vivo Sanger sequencing results (Figure S9) demonstrated that PIO@Lipo also induced POLD4 gene editing in mouse tumor tissues. Quantitative analysis of the editing outcomes using the online Inference of CRISPR Edits (ICE) analysis tool revealed that the Indel frequency reached 28% in vitro cells and 10% in tumor tissues (Figure S10). Subsequently, the mechanistic effects of POLD4 knockdown on cellular functions were investigated. Upon combined treatment with PIO@Lipo and radiotherapy, the ATM‐CHK2‐dependent RAD51 homologous recombination (HR) repair pathway was significantly upregulated in tumor cells. Specifically, the expression levels of P‐ATM were markedly increased in both the radiotherapy group (G5) and the combination therapy group (G6), while its downstream target, P‐CHK2, also showed elevated protein levels. In contrast, the total CHK2 protein expression remained largely unchanged (Figure 5D). RAD51, a core functional protein involved in homologous recombination repair, exhibited a significant upregulation in the G4‐G6, suggesting P‐ATM‐mediated regulation. In addition, POLD4 expression was significantly knocked down in both G4 and G6 groups, demonstrating the successful gene editing ability of PIO@Lipo. Similarly, at the mRNA level, POLD4 expression was significantly downregulated in both G4 and G6 groups (Figure 5E). Notably, partial POLD4 gene editing induced marked mRNA and protein downregulation and the aforementioned radiosensitizing effects, reflecting a threshold‐dependent effect. What's more, RAD51 expression showed significant upregulation in G5 and G6 groups, consistent with the western blot results (Figure 5F). The combination of POLD4 knockdown and radiotherapy treatment triggered a significant upregulation of the ATM‐CHK2 pathway and an elevation of RAD51 levels, demonstrating a robust induction of DNA damage. The ATM‐CHK2 signaling axis serves as the primary sensor and transducer of DNA double‐strand breaks, wherein P‐ATM phosphorylates CHK2 to initiate cell cycle checkpoints and activate DNA repair programs [41]. RAD51, as a central recombinase in homologous recombination, forms nucleoprotein filaments that facilitate strand invasion during DNA repair [42, 43, 44]. The coordinated upregulation of these proteins indicates the presence of extensive DNA damage that surpasses the baseline repair capacity, as evidenced by persistent ATM‐CHK2 activation and RAD51 recruitment. This pattern suggests that POLD4 depletion causes replication fork collapse, synergizing with radiation‐induced DNA breaks. Crucially, this molecular profile confirms the effectiveness of the treatment in inducing irreparable, lethal DNA damage. Although the upregulation of RAD51 reflects a compensatory cellular survival response, the synthetic lethal relationship between POLD4 deficiency and radiotherapy ultimately overwhelms the DNA repair machinery, as demonstrated by the observed reductions in tumor viability and increased levels of apoptosis presented in this study. Furthermore, the intracellular distribution of unencapsulated plasmid and PIO@Lipo was analyzed by fluorescence imaging (Figure 5G). Minimal cellular internalization was observed for unencapsulated plasmids at both 4 and 8‐h time points. In contrast, PIO@Lipo exhibited a distinct intracellular trafficking pattern: at 4 h post‐treatment, partial co‐localization between plasmids (red fluorescence) and lysosomes (green fluorescence) was observed, appearing as yellow signals. At 8 h, the majority of plasmids had escaped lysosomes and entered the nucleus (stained blue by Hoechst), confirming a successful delivery. These results indicate that PIO@Lipo’ quaternary ammonium groups confer a positive surface charge, enabling local membrane disruption to facilitate lysosomal escape.

Despite these favorable properties, the CRISPR system raises significant concerns regarding off‐target editing and consequent genotoxicity. Therefore, this study identified the top 6 potential off‐target sites for the gRNA used via the online Cas‐OFFinder tool (Table S3). Following PCR amplification of products from PIO@Lipo‐mediated gene editing, Sanger sequencing results demonstrated no evidence of gene editing at any of these potential off‐target sites (Figure S11). Additionally, a quantitative assessment of off‐target effects was performed via amplicon sequencing, with off‐target site 1 selected as a representative example. No significant insertions or deletions were detected in the flanking region of this off‐target site, indicating that the CRISPR system did not mediate editing at off‐target loci (Figures S12 and S13).

### 2.5 MRI Performance and Time‐Dependent Biodistribution of PIO@Lipo In Vivo

**FIGURE 6:**
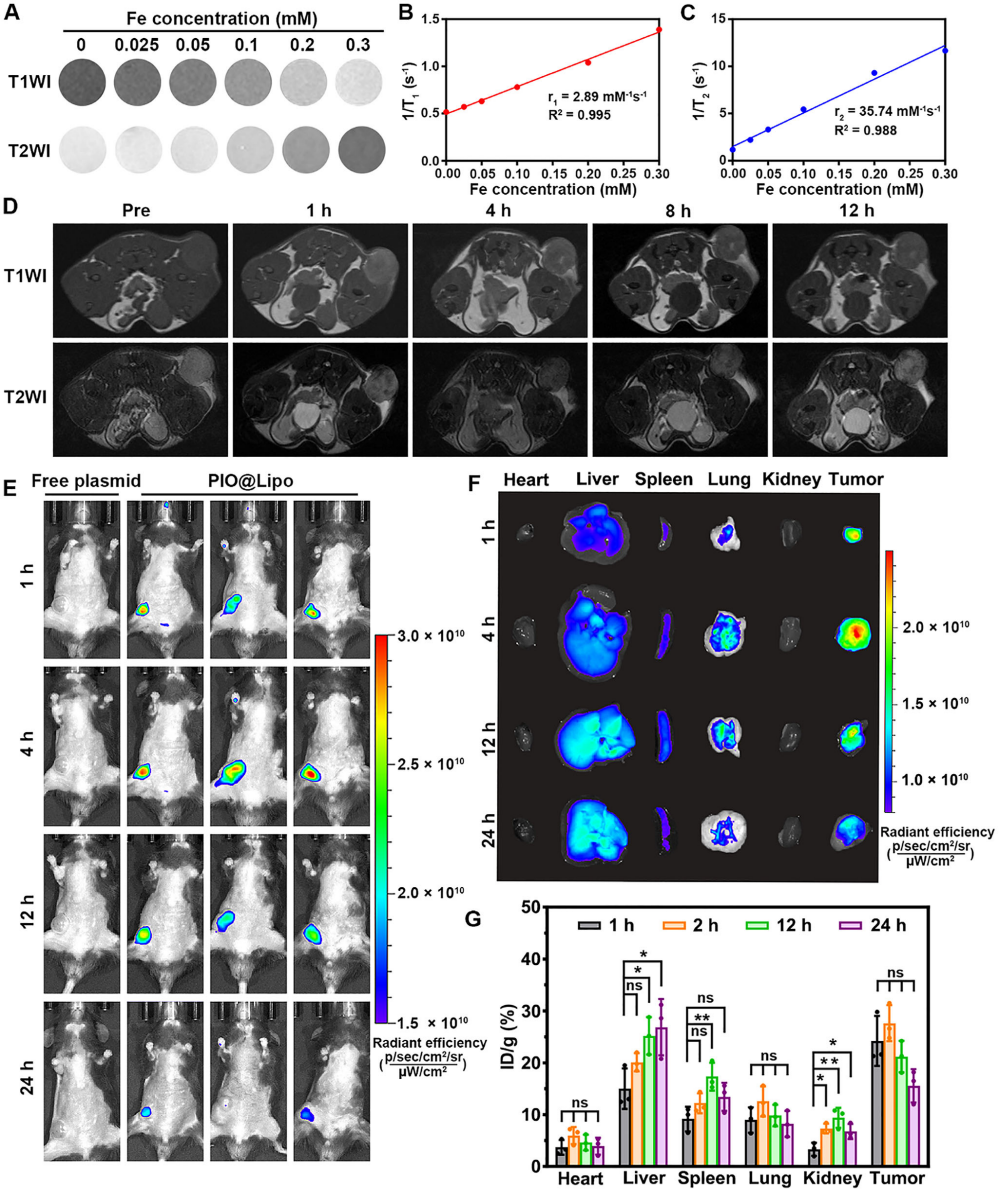
(A) T1‐weighted and T2‐weighted images of PIO@Lipo with different Fe concentrations. B, C) r_1_ and r_2_ values of PIO@Lipo derived from quantitative T1‐weighted and T2‐weighted mapping results. D) Representative T1‐weighted and T2‐weighted images of PIO@Lipo at different time points after tail vein injection. E) In Vivo Imaging System (IVIS) imaging results of fluorescently labeled free plasmids and PIO@Lipo after tail vein injection, with the fluorescent signal in the lower left region indicating the tumor‐bearing site. F) IVIS images showing relative content of PIO@Lipo in various tissues at different time points. G) Quantitative distribution of PIO@Lipo in various tissues determined by ICP‐OES measurement.

Figure 6A displays the MRI results of PIO@Lipo at iron concentrations ranging from 0 to 0.3 mm, which showed dual‐contrast enhancement capability in both T1‐weighted imaging (T1WI) and T2‐weighted imaging (T2WI). Quantitative relaxivity mapping (Figure 6B,C) further indicated that PIO@Lipo had a longitudinal relaxivity (r_1_) of 2.8 mm ^−1^s^−1^ and a transverse relaxivity (r_2_) of 35.7 mm ^−1^s^−1^. The in vivo contrast enhancement capability of PIO@Lipo was further assessed through tail vein intravenous administration (Figure 6D). Prior to I.V. injection (Pre), the tumorous tissue showed comparable signal intensity to adjacent tissues. Post‐injection analysis demonstrated significant signal enhancement in the tumor region from 1‐h post‐administration, with subsequent progressive signal attenuation. At 12 h post‐injection, the enhancement level nearly returned to baseline (Pre‐level). Figure S14 provided a quantitative analysis of tumor enhancement kinetics, highlighting the temporal contrast characteristics of PIO@Lipo. To further investigate the tumor‐targeting capability of PIO@Lipo, iFluor 647‐labeled plasmids were employed for in vivo tracking. As shown in Figure 6E, a clear distinction in biodistribution patterns was observed between free plasmids and PIO@Lipo. While free plasmids showed negligible tumor accumulation and were rapidly cleared from circulation, PIO@Lipo achieved predominant tumor localization within 1‐h post‐administration. The nanoparticle accumulation reached a peak at 4 h, followed by gradual metabolic elimination. By 24 h post‐injection, the concentration of PIO@Lipo in tumor tissue was decreased to less than 50% of its maximum level. Through systematic dissection of mouse models, the biodistribution profile of PIO@Lipo across major organ systems was investigated (Figure 6F). Following tail vein injection, PIO@Lipo demonstrated significant tumor accumulation at both 1 and 4‐h timepoints, with peak enrichment intensity observed at 4 h. Importantly, tumor accumulation levels were higher than those detected in the lung, liver, and spleen, thereby confirming the effective and selective tumor‐targeting capability mediated by the EPR effect. By 12 h post‐injection, nanoparticle levels in tumor tissue began to decrease, while accumulation in the liver and spleen continued to rise progressively. At the 24‐h endpoint, nanoparticle concentrations in the tumor were decreased below those in the liver and spleen, indicating that these organs served as the primary routes of nanoparticle clearance. To quantitatively analyze nanoparticle distribution across organs, this study employed Inductively Coupled Plasma Optical Emission Spectroscopy (ICP‐OES) to measure iron content in various tissues (Figure 6G). Consistent with IVIS observations, ICP‐OES quantification revealed that nanoparticle accumulation in tumor tissue reached its peak at 4 h post‐injection, followed by a gradual decline at 12 and 24 h. In contrast, accumulation in the liver and spleen was increased progressively over 24 h, while minimal deposition was detected in the heart, lung, and kidney. In the present study, the higher accumulation of PIO@Lipo in tumors compared to the liver and lungs within the first 4 h post‐intravenous injection can be attributed to the following factors. First, the PEGylation ratio of the liposomes is 3%, which is higher than the conventional 1.5%. This elevated pegylation confers a strong steric shielding effect that significantly prolongs the in vivo circulation time [45]. Second, zeta potential measurements confirm that PIO@Lipo has a surface charge of 15.4 mV, which is much lower than the >50 mV reported for conventional DOTAP‐based liposomes in the literature [46]. This moderate cationic charge avoids excessive electrostatic interactions with plasma albumin and red blood cells, and reduces undesired retention within the liver and lungs [47]. Additionally, RM‐1 prostate cancer cells exhibit rapid proliferation, developing an abundant capillary network within the tumor. Moreover, prostate cancer tissues highly express the intercellular adhesion molecule‐1 (ICAM‐1), which in turn promotes the recruitment of neutrophils [48]. Subsequent neutrophil degranulation releases elastase, disrupting the vascular endothelial barrier and inducing a robust EPR effect [49]. The EPR effect is attributed to structural abnormalities in tumor vasculature, which contains enlarged fenestrations that facilitate the accumulation of nanoparticles in tumor tissues [50]. Additionally, the primary clearance pathways for PIO@Lipo involve hepatic and splenic filtration through the RES, where specialized phagocytic populations mediate nanoparticle recognition and internalization, which is precisely why accumulation in the liver and spleen is observed at 12 h post‐injection. Given their hydrodynamic diameter exceeding the renal filtration threshold, urinary excretion is virtually negligible.

### 2.6 Evaluation of Therapeutic Efficacy of PIO@Lipo

**FIGURE 7:**
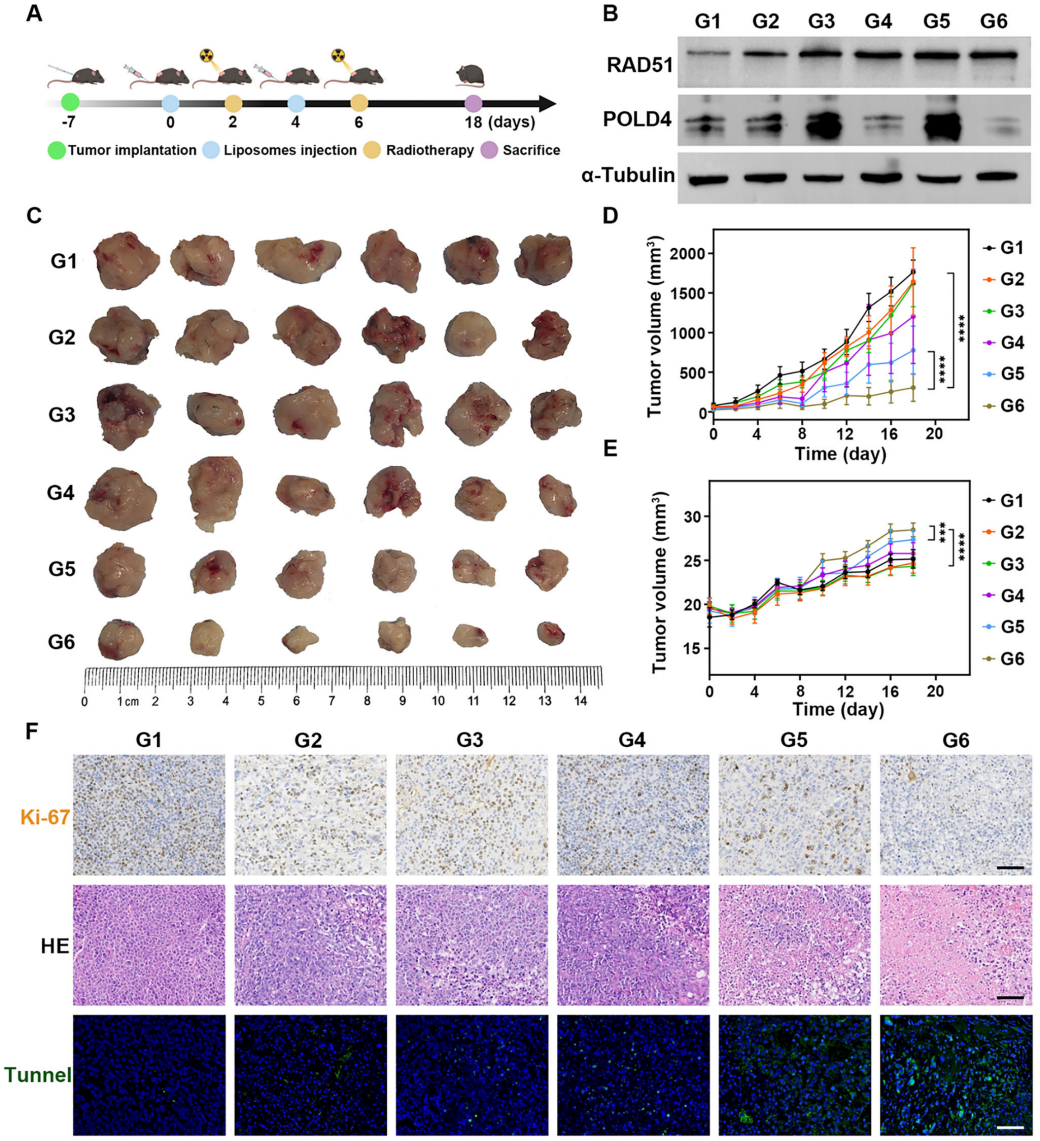
(A) Experimental procedures and time points for mice in this study. B) Western blot analysis of RAD51 and POLD4 expression in the tumor tissues. C) Gross appearance of dissected tumor tissues from mice. D, E) Changes in tumor size and body weight of mice over time. F) Results of Ki67, HE, and Tunnel staining in tumor tissues from each experimental group (scale bar: 100 µm).

Figure 7A illustrates the detailed animal experimental protocol. After tumor inoculation, mice received tail vein injections of nanomaterials followed by radiotherapy every two days for two treatment cycles. In this study, PIO@Lipo was administered 2 days prior to the first radiotherapy to allow effective POLD4 knockdown. On day 18, the mice were euthanized for therapeutic efficacy analysis. Throughout the study period, tumor volume and body weight were recorded every two days. Following euthanasia, tumor tissues were collected and subjected to total protein extraction. The expression levels of POLD4 and RAD51 in the tumor tissues were then analyzed (Figure 7B). The results revealed that RAD51 expression was significantly upregulated in treatment groups G3‐G6 compared to the control group, with the most pronounced increase in G5‐G6 groups. POLD4 expression analysis confirmed successful knockdown in G4 and G6 groups, whereas the G5 group showed elevated POLD4 levels, which may be attributed to radiation‐induced reactive upregulation. Figure 7C displays the gross specimens of excised tumors; the G5 and G6 groups showed significantly smaller tumor masses compared to other groups, with G6 exhibiting the most pronounced reduction in tumor volume. Notably, the tumor tissues in G6 appeared markedly paler in color, indicating concurrent inhibition of intratumoral angiogenesis. Figure 7D presents the quantitative results of tumor growth over time. The G6 group presented significantly slower tumor progression compared to both G1 and G5 groups (both *p* < 0.0001), indicating a potent inhibitory effect on tumor growth. Additionally, mice in the G6 combination therapy group showed stable weight gain, with significantly higher body weight observed compared to the G5 group (*p* = 0.002) and other groups (all *p* < 0.0001, Figure 7E). These results illustrated that the combined gene therapy and radiation treatment effectively mitigated tumor‐induced wasting and malnutrition in the mice. Histopathological analysis of tumor sections revealed significant alterations in malignancy (Figure 7F). Ki‐67 staining showed a high proportion of yellow Ki‐67‐positive cells across G1‐G4 groups, indicating active tumor proliferation. In contrast, the number of Ki‐67‐positive cells was markedly decreased in G5‐G6 groups, with the lowest level observed in G6. Hematoxylin and Eosin (HE) staining showed that untreated tumors exhibited large, hyperchromatic nuclei, while combined radiotherapy and gene therapy resulted in nuclear pyknosis, fragmentation, and an increased cytoplasmic ratio. Furthermore, the Tunnel assay revealed minimal apoptotic activity in the G1‐G4 groups, as evidenced by few Tunnel‐positive cells. A moderate increase was observed in G5, with a significant elevation in G6, thereby confirming enhanced tumor cell apoptosis. In vivo studies demonstrated that targeted POLD4 knockdown synergistically enhanced the efficacy of radiotherapy in prostate cancer. Mechanistically, this therapeutic approach compromised genomic stability by impairing DNA damage repair and activating programmed cell death pathways. The intervention not only suppressed radioresistance networks but also significantly attenuated malignant progression. Thus, this study provides evidence that POLD4 serves as an effective radiosensitization target, offering new therapeutic potential for radiotherapy enhancement.

### 2.7 Immunomodulation and Biocompatibility of PIO@Lipo‐Mediated Radiotherapy

**FIGURE 8:**
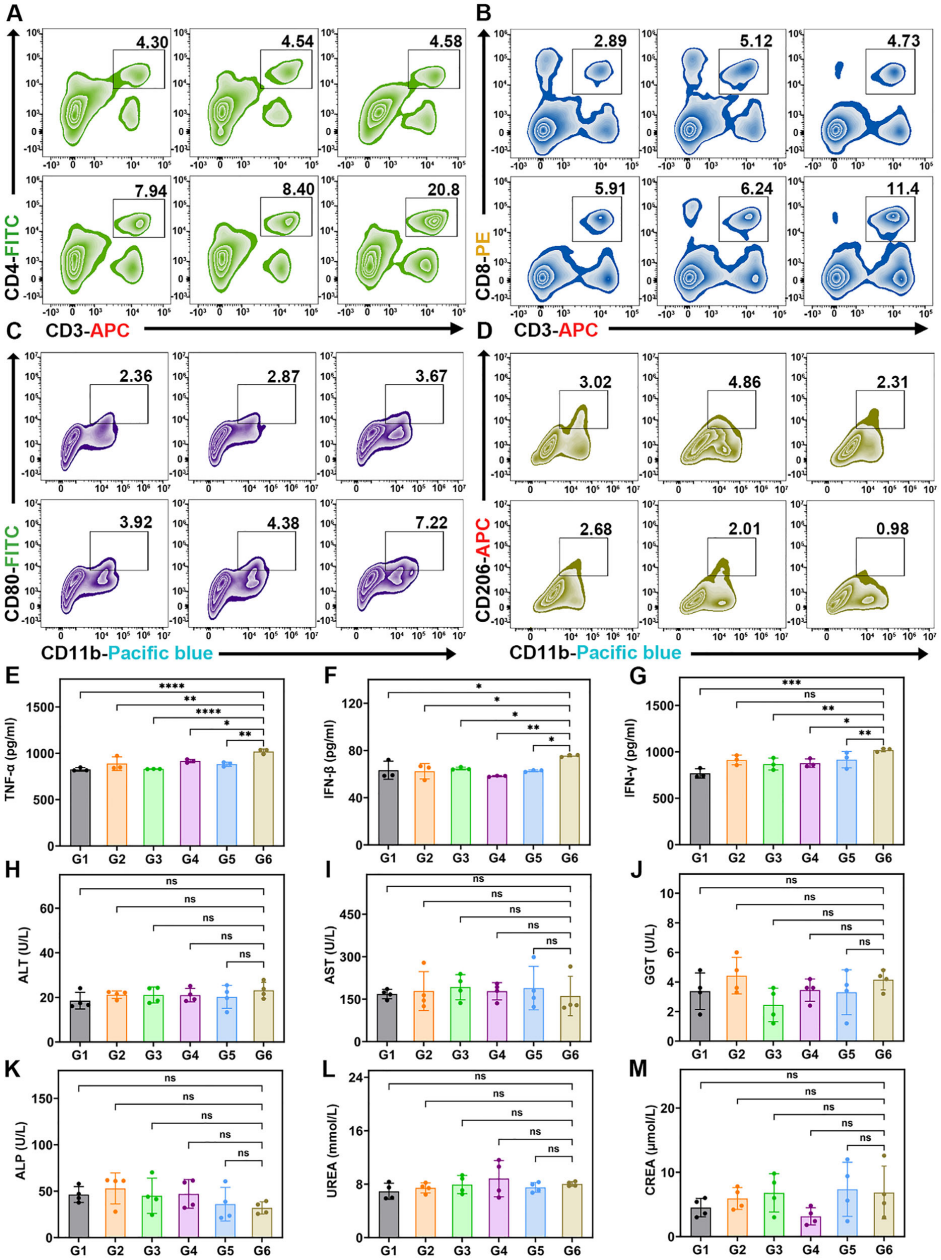
(A, B) Flow cytometry analysis of T cells in splenic tissues from each group, with boxes and numbers above indicating the percentages of CD4^+^ and CD8^+^ T cells. C, D) Flow cytometry analysis of macrophages in tumor tissues, with boxes and numbers above indicating the percentages of M1 (CD80) and M2 (CD206) cells. E–G) ELISA results of inflammatory factors in tumor tissues. H–M) Biochemical results of six liver and kidney function parameters in mouse serum.

Through enzymatic digestion of spleens and tumor tissues obtained from treated mice, flow cytometric analysis was performed to quantitatively assess the infiltration levels of various immune cell subsets. Figure 8A,B shows the dynamic alterations in CD4^+^ and CD8^+^ T cell populations within splenic tissues following therapeutic intervention. Quantitative evaluation revealed a progressive increase in CD4^+^ T cell proportions in splenic tissues across the treatment groups, escalating from 4.3% in G1 to 8.4% in G5, with a peak of 20.8% in G6. Similarly, CD8^+^ T cell populations showed a corresponding increase, rising from a baseline of 2.8% to 11.4% in G6. Collectively, these quantitative shifts indicated a strong activation of the host immune system in response to the therapeutic intervention. Macrophage infiltration in tumor tissues was further investigated. As shown in Figure 8C, the proportion of pro‐inflammatory M1 macrophages (CD80^+^) is significantly increased from 2.3% in G1 to 7.2% in G6. In contrast, the percentage of immunosuppressive M2 macrophages (CD206^+^) is decreased from 3.0% to 0.9%, as depicted in Figure 8D. This shift in macrophage polarization indicates a remodeling of the tumor microenvironment, favoring enhanced anti‐tumor immunity through increased infiltration of tumoricidal immune cells. Furthermore, ELISA analysis of cytokines in tumor tissues (Figure 8E–G) demonstrated that the combination therapy group (G6) exhibited significantly higher levels of anti‐tumor cytokines, including TNF‐α, IFN‐β, and IFN‐γ, compared to other treatment groups (G1‐G5). These findings suggest that the synergistic effect of radiotherapy and gene therapy markedly enhances the production of key immunostimulatory cytokines, which contribute to the observed anti‐tumor immune responses. These experimental results support that the combination of radiotherapy and gene therapy induces significant immunogenic cell death (ICD) in tumor cells, leading to the release of damage‐associated molecular patterns (DAMPs) and tumor‐associated antigens. These molecules subsequently promote the maturation of antigen‐presenting cells, such as dendritic cells, and ultimately activate both CD4^+^ helper T cells and CD8^+^ cytotoxic T cells [51]. Furthermore, the activated CD4^+^ and CD8^+^ T cells secrete cytokines such as IFN‐γ and TNF‐α, which promote the polarization of tumor‐associated macrophages toward the M1 phenotype, thereby transforming immunologically “cold” tumors into “hot” tumors with enhanced immune infiltration and improved therapeutic responsiveness [52, 53]. For evaluating the biosafety of PIO@Lipo, six blood biochemical parameters associated with liver and kidney function across all experimental groups were assessed (Figure 8H–M). The results revealed no statistically significant alterations in these indicators, indicating that PIO@Lipo does not induce detectable hepatotoxicity or nephrotoxicity in vivo. Moreover, histopathological examination of major organs, including the heart, liver, spleen, lungs, and kidneys, in treated mice, presented no significant morphological abnormalities post‐treatment, further confirming the negligible organ toxicity (Figure S15). To further evaluate potential systemic effects, a 10‐parameter complete blood count was performed (Figure S16). While the majority of hematological indices remained within normal ranges across all groups, a slight decrease in white blood cell and neutrophil counts was observed specifically in G5 and G6 groups, a phenomenon likely attributable to the effects of radiation therapy.

## 3 Conclusion

This study has proposed an innovative MRI‐monitored gene therapy platform for prostate cancer, developed by co‐encapsulating CRISPR/Cas9 plasmids and USPIONs within cationic liposomes. Through systematic in vitro and in vivo evaluations, it has been demonstrated that the resulting PIO@Lipo, comprised of CRISPR/Cas9 plasmids and USPIONs, can significantly improve the therapeutic efficacy of radiotherapy through targeted knockdown of POLD4. The mechanistic investigations further revealed that PIO@Lipo effectively promotes tumor cell apoptosis and remodulates the immunosuppressive tumor microenvironment. These findings collectively establish POLD4 as a viable molecular target for enhancing radiosensitivity in prostate cancer, offering a promising therapeutic strategy with potential for future clinical translation.

## 4 Experimental Section

### 4.1 Reagents and Materials

The lipid components, including 1,2‐dioleoyl‐3‐trimethylammonium‐propane (DOTAP, Cat# D351073), 1,2‐dioleoyl‐sn‐glycero‐3‐phosphocholine (DOPC, Cat# D130438‐1g), and 1,2‐distearoyl‐sn‐glycero‐3‐phosphoethanolamine‐N‐[amino(polyethylene glycol)‐2000] (DSPE‐PEG‐NH_2_, Cat# D130802), along with cholesterol (Cat# C432976), were procured from Aladdin Biochemical Technology Co., Ltd. (Shanghai, China). Chloroform (Cat# 31000500112) was supplied by Jiangsu Qiangsheng Functional Chemical Co., Ltd. (Jiangsu, China). Iron(III) acetylacetonate (Fe(acac)_3_, Cat# F300), oleic acid, and oleylamine were purchased from Sigma–Aldrich Co. (USA). Isopropanol, acetone, ethanol, cyclohexane, and tetrahydrofuran (THF) were obtained from Sinopharm Chemical Reagent Co., Ltd. (Hangzhou, China). Bisphosphonate‐PEG (DP‐PEG) was provided by Xinying Biomedical Technology Co., Ltd. (Suzhou, China). The murine prostate cancer cell line RM‐1 (RRID: CVCL_B459, Cat# CL‐0198) was purchased from Procell Life Science & Technology Co., Ltd. (Wuhan, China) on March 1, 2024. Manufacturer‐conducted STR profiling confirmed a complete match with RM‐1 reference standards, with additional certification of being free from bacterial, fungal, and mycoplasma contamination. All experimental procedures followed standard sterile protocols with maintained cell viability, thereby safeguarding the reliability of research conclusions. The Roswell Park Memorial Institute (RPMI)‐1640 medium, fetal bovine serum (FBS), penicillin, and streptomycin were supplied by HyClone (UT, USA). 6–8 weeks old male C57BL/6J mice were purchased from Changzhou Cavins Model Animal Co., Ltd. The experimental protocols involving animals were approved by the Institutional Animal Care and Use Committee (IACUC) of Soochow University (202410A0306).

### 4.2 Statistics Analysis

Statistical analyses were performed using GraphPad Prism 10.0.2 software. One‐way analysis of variance (ANOVA) with Dunnett's post hoc test for multiple comparisons was applied to assess intergroup differences (statistical significance threshold: *p* < 0.05). Data from three or more independent experiments were expressed as mean ± SD, with significance levels denoted as ^*^ *p* < 0.05, ^**^ *p* < 0.01, ^***^ *p* < 0.001, and ^****^ *p* < 0.0001, and “ns” indicating non‐significance.

For more experimental details, including the synthesis of nanomaterials, material characterization, and cellular/animal biology experiments could be found in the Supporting Information.

## Conflicts of Interest

The authors declare no conflicts of interest.

## Supporting information




**Supporting File**: advs73727‐sup‐0001‐SuppMat.docx.

## Data Availability

The data that support the findings of this study are available from the corresponding author upon reasonable request.
