# Supplementary material for: CRISPR‐Cas9‐Loaded Theranostic Liposomes for Enhancing Radiosensitization of Prostate Cancer through POLD4 Gene Editing under Real‐Time MRI Monitoring
Source: Adv Sci (Weinh). 2026 Jan 7;13(16):e19704. doi: 10.1002/advs.202519704 (PMC13042682; doi:10.1002/advs.202519704)
Supplement: Supplementary file 1 — Supporting File: advs73727‐sup‐0001‐SuppMat.docx. [file ADVS-13-e19704-s001.docx]

**CRISPR-Cas9-Loaded Theranostic Liposomes for Enhancing Radiosensitization of Prostate Cancer through POLD4 Gene Editing under Real-Time MRI Monitoring**

**Xuhui Fan^1^, Ruru Zhang^2^, Linjun Yang^3^, Shixiong Chen^1^, Meijuan He^1^, Yongqiang Wang^1^, Linjie Huang^1^, Jianfeng Zeng^2^, Shuwang Wu****^2^, Mingyuan Gao^2,4*^, Han Wang^1,5,6*^**

^1^Department of Radiology, Shanghai General Hospital, Shanghai Jiao Tong University School of Medicine, Shanghai 200080, R. P. China

^2^State Key Laboratory of Radiation Medicine and Protection, School for Radiological and Interdisciplinary Sciences (RAD-X), Collaborative Innovation Center of Radiation Medicine of Jiangsu Higher Education Institutions, Soochow University, Suzhou 215123, P. R. China

^3^Department of Bone and Joint Surgery, Renji Hospital, Shanghai Jiao Tong University School of Medicine, Shanghai 200001, P. R. China.

^4^School of Life Sciences, Suzhou Medical College, Soochow University, Suzhou 215123, P. R. China

^5^Shanghai General Hospital Branch of National Center for Translational Medicine (Shanghai), Shanghai 201620, R. P. China

^6^Jiading Branch of Shanghai General Hospital, Shanghai 201803, R. P. China

**^*^Correspondence to** Han Wang, **email**: [han.wang@shsmu.edu.cn](mailto:han.wang@shsmu.edu.cn); Mingyuan Gao, **email**: [gaomy@ suda.edu.cn](mailto:gaomy@iccas.ac.cn)  **Experimental Section**

*Transcriptome Sequencing:* RM-1 cells were divided into irradiation-treated (6 Gy X-ray, n=3) and control (n=3) groups. Total RNA was extracted and mRNA enriched using Oligo(dT) magnetic beads. Following fragmentation, cDNA libraries were prepared via reverse transcription with random hexamers, second-strand synthesis, and AMPure XP bead purification. After end repair, A-tailing, and adapter ligation, size-selected fragments were PCR-amplified. Quality-controlled libraries were pooled and sequenced on a second-generation Illumina platform using PE150 strategy. Differential gene expression analysis was performed with DESeq (*p*adj < 0.05), followed by GO enrichment (biological process, molecular function, cellular component) and KEGG pathway analysis using clusterProfiler. The comparative analysis of POLD4 expression between normal and prostate cancer issues was performed using publicly available RNA-seq data from The Cancer Genome Atlas (TCGA)-PRAD (Prostate Adenocarcinoma) cohort.

*Plasmid Construction and Amplification:* The POLD4 plasmid was commercially obtained from Genomeditech Co., Ltd. For gRNA-directed targeting, single-stranded DNA oligonucleotides encoding the gRNA sequence were chemically synthesized, followed by annealing to generate double-stranded DNA adapters. These oligos were designed with flanking restriction enzyme sites for directional cloning into the linearized CRISPR/Cas9 backbone vector (PX458) via T4 DNA ligase (Fermentas). The ligation products were transformed into chemically competent E. coli cells. Transformed bacteria were plated on antibiotic-containing agar, spread evenly with a sterile spreader, and incubated overnight at 37°C. Selected colonies were cultured for plasmid extraction (DP107, TIANGEN), screened by colony PCR, and confirmed via Sanger sequencing. Only clones demonstrating 100% sequence identity to the designed gRNA template were selected for plasmid amplification and purification. The sgRNA-resistant POLD4 cDNA plasmid was commercially synthesized by Genewiz Suzhou Co., Ltd. This plasmid harbors a synonymous mutation-modified POLD4 cDNA that confers resistance to sgRNA targeting without altering the encoded amino acid sequence. The mutant POLD4 cDNA (flanked by *HindIII/XhoI* sites) was digested, ligated into linearized pCDNA3.1, and processed through transformation, antibiotic selection, colony screening, and Sanger sequencing validation, following the identical procedures described for the PX458 plasmid construction above. For plasmid large-scale extraction, E. coli harboring the target plasmid were cultured in 300 mL LB medium (Sangon Biotech) at 37°C with 250 rpm shaking for 16 h. Bacterial cells were harvested and lysed following the manufacturer's protocol (DP117, TIANGEN), followed by plasmid purification using column-based purification. The purified plasmids were quantified via spectrophotometry (Nanodrop 2000c, Thermo) and stored at -20°C until use.

*Agarose Gel Electrophoresis and T7E1:* Genomic DNA was extracted from CRISPR/Cas9-edited cells using the DP304 DNA Extraction Kit (TIANGEN). The POLD4 target region was amplified by PCR with Phanta UniFi DNA Polymerase (P516, Vazyme) under manufacturer-recommended conditions. PCR products were mixed with 6×DNA Loading Buffer (Beyotime) and electrophoresed on 1.5% agarose gels (precast gels, Beyotime) in 1×TAE buffer at 120 V for 30 min. To verify CRISPR/Cas9-induced mutations, PCR-amplified target fragments were mixed with T7E1 Reaction Buffer (EN303, Vazyme). The mixture was denatured at 95°C for 5 min, followed by annealing using a manufacturer's gradient cooling protocol to facilitate heteroduplex formation. After adding 1 μL T7 Endonuclease I, samples were incubated at 37°C for 15 min. Digestion products were analyzed by 2% agarose gel electrophoresis (Beyotime), with indel efficiency based on cleaved band intensity.

‌*Synthesis of USPIONs:* Fe(acac)₃ was purified by double recrystallization prior to use. In a typical synthesis, the reaction mixture containing Fe(acac)₃ (20 mmol), oleic acid (120 mmol), and oleylamine (120 mmol) in 0.9 L isopropanol was prepared under nitrogen purging for 10 minutes. Then transferred into a stainless-steel tubular reactor at 270°C using a high-pressure pump (flow rate: 30 mL/min, pressure: 5.2 MPa) for pyrolysis. The resulting nanoparticles were collected by precipitation. For purification, the hydrophobic Fe₃O₄ nanoparticles underwent three cycles of redispersion in cyclohexane and precipitation with acetone. For surface functionalization, a mixture of 50 mg hydrophobic nanoparticles and 500 mg DP-PEG in 10 mL THF was prepared by sonication. The reaction mixture was heated to 40°C with continuous stirring for 48 h. The PEGylated nanoparticles were then precipitated with cyclohexane, washed three times by centrifugation, and vacuum-dried. The final hydrophilic nanoparticles were obtained by dissolving the product in water, followed by three rounds of ultrafiltration using 30 kDa MWCO centrifugal filters to remove excess PEG ligands.

*Synthesis of PIO-Lipo:* The *PIO-Lipo* were prepared using a modified thin-film hydration technique. Briefly, DOTAP, DOPC, cholesterol, and DSPE-PEG-NH_2_ at a molar ratio of 50:10:37:3 were co-dissolved in 3 mL chloroform within a round-bottom flask. The organic solvent was then completely evaporated under reduced pressure using a rotary evaporator at 37°C to form a thin lipid film on the flask wall. After vacuum drying overnight to remove residual solvent, a homogeneous aqueous suspension (3 mL) containing plasmid (plasmid-to-lipo mass ratio 1:3,mixed with protamine sulfate to facilitate nuclei-targeted deliver) and USPIONs was introduced into the lipid film-coated flask. The resulting mixture was immediately subjected to bath sonication (40 kHz, 100 W) in an ice-water bath for 5 min to ensure uniform nanoparticle dispersion. Subsequently, the suspension was processed through 10 consecutive extrusion cycles using an Avanti Polar Lipids mini-extruder equipped with a 100 nm polycarbonate membrane to obtain monodisperse nanoparticles. The obtained nanoparticles were further purified by dialysis against deionized water using a nanoporous membrane (30 nm pore size) for 12 h with three buffer exchanges.

*Gel Retardation Assay:* To determine the optimal mass ratio of liposomes to plasmid DNA, gel retardation assays were performed. Plasmid DNA was mixed with liposomes at varying mass ratios and incubated at room temperature for 30 min. After adding 6×DNA Loading Buffer, samples were loaded onto a 1.0% agarose gel (Beyotime) alongside free plasmid and DNA Marker D0117S (Beyotime). Electrophoresis was conducted in 1×TAE buffer at 120 V for 30 min. The optimal liposomes ratio was identified as the minimum ratio at which plasmid DNA remained fully retained in the loading well, indicating complete complexation.

*PicoGreen Assay:* The encapsulation efficiency (EE%) of plasmid-loaded liposomes was quantified using the PicoGreen dsDNA Quantitation Kit (AC13850-1ml, ACMEC). A standard curve (0.625 - 100 ng/ml) was prepared per the manufacturer’s instructions using calf thymus dsDNA. For total plasmid measurement, liposome suspension was treated with Triton X-100 to disrupt membranes and release encapsulated plasmids. Free plasmids were collected from the dialyzed external solution. 100 μl samples were mixed with 100 μl diluted PicoGreen reagent (1:200 in TE buffer), incubated in the dark for 5 minutes at room temperature, and fluorescence was measured at 488 nm (excitation) and 520 nm (emission) using a microplate reader. Total (C_total) and free (C_free) plasmid concentrations were derived from the standard curve. EE% was calculated as: EE (%) = [(C_total - C_free)/C_total] × 100%.

*Characterization:* The hydrodynamic diameter and zeta potential of nanoparticles were measured using a Zetasizer Nano ZS90 (Malvern Panalytical) equipped with a high-stability He-Ne laser. XPS analysis was performed on a Thermo Scientific K-ALPHA spectrometer with Al Kα radiation (1486.6 eV), and binding energies were calibrated against the C 1s peak at 284.8 eV. FTIR spectra were recorded on a Thermo Nicolet IS5 spectrometer in the range of 400–4000 cm⁻¹, using KBr pellet method. The morphology of nanomaterials was examined using a Thermo Scientific Talos F200S transmission electron microscope operated at 200 kV. Samples were prepared by depositing nanoparticle suspensions onto standard copper grids and air-dried at room temperature. For liposome samples, a 3% phosphotungstic acid (P769541, Macklin) solution was added for negative staining to enhance contrast. Elemental distribution analysis was performed with an energy-dispersive X-ray spectroscopy (EDS) system equipped with SUPER X detectors.

*Cck8:* Cells were seeded in 96-well plates at a density of 3000 cells/well and cultured for 24 h prior to treatment. PIO@Lipo (plasmid concentration: 1 μg/mL) or equimolar concentrations of other lipid-based nanomaterials were added to the wells and incubated for 48 h. After removing the supernatant, cells were washed once with PBS, followed by the addition of 100 μL fresh medium containing 10% CCK-8 reagent (Fude Biology). Absorbance was measured at 562 nm using a Synergy 2 microplate reader (BioTek Instruments, USA). The cell viability (%) is calculated by dividing the absorbance values of experimental groups by those of the untreated controls, with background signals from the culture medium alone subtracted.

‌*Colony Formation Assay:* Cells were seeded in 6-well plates at a density of 500 cells per well and allowed to adhere for 24 h. Following attachment, cells were subjected to radiation treatment and/or nanoparticle exposure. After 10 days of culture, the medium was aspirated, and cells were gently washed once with PBS. Fixation was performed with 4% paraformaldehyde (Biosharp) for 5 min at room temperature. Subsequently, cells were stained with crystal violet solution (Beyotime) for 10 min, followed by three PBS washes to remove excess dye. Colonies were photographed under bright-field microscopy and quantified.

*Live/dead Cell Staining:* Cells were seeded in confocal dishes (Biosharp) at a density of 3×10⁴ cells/well and cultured for 24 hours prior to treatment, followed by incubation with nanomaterials (plasmid at 1 μg/mL) and X-ray irradiation. After 48-hour post-treatment, cells were stained using the Calcein AM/PI Double Staining Kit (Beyotime, C2015S) according to the manufacturer’s protocol: briefly, cells were washed once with PBS (pH 7.4), incubated with freshly prepared Calcein AM/PI working solution for 30 minutes at 37°C in the dark, and immediately visualized under an Olympus FV1200 confocal microscope (Japan) with excitation/emission filters set at 490/517 nm (green fluorescence, Calcein AM for live cells) and 535/617 nm (red fluorescence, PI for dead cells).

*Apoptosis Flow Cytometry:* Cells were seeded in 6-well plates and incubated for 6 hours prior to treatment with nanomaterials and radiation therapy. After 48-hour incubation, all cells (including floating cells in the supernatant) were harvested by trypsinization, centrifuged, washed once with PBS, and resuspended in Annexin V Binding Buffer from the Elabscience (E-CK-A218). Subsequently, cells were stained with Annexin V-APC and 7-AAD for 20 minutes in the dark at room temperature. After a final PBS wash, samples were analyzed immediately using flow cytometry with the following configurations: Annexin V-APC signal collected through the APC channel (Ex/Em 650/660 nm) and 7-AAD through the PerCP-Cy5.5 channel (Ex/Em 488/670 nm).

*Western Blot:* cells were cultured in 10 cm dishes and treated with nanomaterials combined with radiation therapy for 48 hours, followed by trypsinization and PBS washing. Cell pellets were lysed with 200 μL of moderate-strength RIPA buffer (FD008, Frode Biological) on ice for 15 minutes, centrifuged at 13,000 rpm for 10 minutes to remove debris, and mixed with 5X loading buffer (FD006, Frode Biological). After boiling at 100°C for 5 minutes, samples were loaded onto a 4-20% polyacrylamide precast gel (P0821S, Beyotime) for electrophoresis and transferred to a 0.22 μm PVDF membrane (ISEQ00010, Millipore). The membrane was blocked with rapid protein-free blocking buffer (PS108P, Yeasen) for 30 minutes, washed once with TBST, and incubated with primary antibodies at 4°C for 12 hours. After secondary antibody incubation (Frode Biological) for 1 hour and final TBST washes, protein signals were detected using ECL substrate (FD8000, Frode Biological) on a Tanon 5200 imaging system. The primary antibodies used were: α-Tubulin (AF2827, Beyotime), Bcl-2 (AF6285, Beyotime), Bax (AB026, Beyotime), cleaved-caspase3 (AC033, Beyotime), and γH2AX (AF5836, Beyotime), POLD4 (YS0004, Immunoway), P-ATM (AF8225, Affinity), RAD51 (DF8066, Affinity), P-CHK2 (AF3036, Affinity), and CHK2 (AF6033, Affinity).

*Quantitative PCR:* Total RNA was extracted from both raw materials and 48h-post-irradiation cells using RNA Extraction Kit (RC102-01, Vazyme) following the manufacturer's protocol, then reverse-transcribed into cDNA with Reverse Transcription Kit (R433-01, Vazyme), whereafter qPCR was performed using cDNA templates mixed with SYBR Green Master Mix (Q412, Vazyme) and gene-specific primers in 20 μL reaction volumes (supplemented with DEPC water), with quantitative analysis conducted on a ViiA-7 Real-Time PCR System (Life Technologies).

*Sanger Sequencing:* Genomic DNA was isolated from CRISPR/Cas9-edited cells using the Genomic DNA Extraction Kit (DP304, Tiangen) according to the manufacturer’s protocol, followed by PCR amplification of the POLD4 target region using gene-specific primers. The amplified products were purified via enzymatic cleanup and subjected to bidirectional Sanger sequencing on an ABI 3730xl platform (Genewiz Suzhou Co., Ltd.). To verify editing efficiency, the sequencing results were analyzed using the web-based platform of EditCo Bio, Inc. (https://ice.editco.bio/#/), where the aligned wild-type and edited sequences were assessed for gene discordance.

*Off-Target Site Search and Amplicon Sequencing:* Potential off-target sites of the designed gRNA were predicted using the Cas-OFFinder online tool. The top 6 candidate sites with the highest homology to the target sequence were selected for validation. Amplicons spanning these candidate off-target regions were generated via PCR with specific primers, and amplicon sequencing was commissioned to Genewiz Suzhou Co., Ltd. to confirm the absence of unintended gene editing events.

*Plasmid Fluorescence Labeling and Lysosomal Escape Assays:* Plasmids were biotinylated PHOTOPROBE® (Long Arm) Biotin (SP-1020, Vector Laboratories) according to the manufacturer’s protocol, followed by incubation with iFluor® 647-streptavidin conjugate (16966, AAT Bioquest) for 1 hour at room temperature in the dark to achieve red fluorescent labeling via biotin-streptavidin binding. Cells were seeded into confocal dishes and transfected with red fluorescent-labeled plasmids, followed by nuclear staining with Hoechst 33342 (C1017, Beyotime) and lysosomal staining with Lyso-Tracker Green (C1047S, Beyotime) according to the manufacturer’s protocols. Live-cell imaging was performed using the confocal microscope at 4 h and 8 h post-transfection to monitor intracellular trafficking and lysosomal escape dynamics.

*Tumor-bearing Mouse Model Establishment:* C57BL/6J mice were housed in a specific pathogen-free (SPF) facility with a 12-h light/dark cycle and relative humidity of 40–70%. At 8 weeks of age, the skin on the lateral right hind limb was depilated (1.5 cm diameter). 50 μL PBS-based RM-1 cell suspension containing 1.5×10⁶ RM-1 cells was subcutaneously injected into the depilated area. Tumor size and mouse body weight were monitored every two days. Subsequent experiments were initiated on day 7 post-inoculation when the tumor volume reached approximately 100 mm³.

*MRI Performance of PIO@Lipo:* PIO@Lipo were prepared in tubes at iron concentrations ranging from 0 to 0.3 mM, and T1WI and T2WI were performed on a 3.0 T MRI scanner (MRS-3031, MR Solutions), followed by quantitative mapping to calculate the longitudinal (r₁) and transverse (r₂) relaxivities through linear regression of the inverse relaxation times (1/T₁ and 1/T₂) against iron concentration. For in vivo studies, PIO@Lipo were intravenously administered to tumor-bearing mice, and T1WI/T2WI scans of the tumor region were performed at 1, 4, 8, 12, and 24 h post-injection, with the signal-to-noise ratio (SNR) quantified as the mean signal intensity of the tumor divided by the standard deviation of background noise.

‌ *In Vivo IVIS Imaging‌:* free iFluor® 647-labeled plasmids and plasmid-loaded PIO@Lipo were intravenously injected into mice via the tail vein, and whole-body fluorescence imaging was performed at 1, 4, 12, and 24 h post-injection using an IVIS Lumina XR III system (PerkinElmer). Subsequently, mice were sacrificed at each time point, followed by ex vivo imaging of excised heart, liver, spleen, lungs, kidneys, and tumor tissues to analyze the plasmid accumulation in different organs.

*ICP-OES Analysis:* Major organs from PIO@Lipo-injected mice were dissected, and tissue samples were digested with nitric acid followed by dilution with water. The Fe concentration in digested solutions was quantified using a Thermo Scientific iCAP 7200 spectrometer, and PIO@Lipo distribution in each tissue was calculated using the formula: [(measured concentration × digestion solution volume) × (total organ mass ÷ partial organ mass)] ÷ injected dose ÷ total organ mass × 100.

*H&E Staining:* Tumor tissues and organs were dissected and fixed in 4% paraformaldehyde, then processed through paraffin embedding, sectioning at 4-5 μm thickness, deparaffinization with xylene, standard H&E staining, graded ethanol dehydration, and finally whole-slide digital scanning using a high-resolution slide scanner (KF-FL-020, KFBIO) to obtain H&E-stained tissue sections.

*IHC and Tunnel Staining:* Tissues underwent identical initial processing as HE staining. Ki67 detection involved heat-mediated antigen retrieval in citrate buffer, peroxidase blocking with 3% H₂O₂, and serum blocking before overnight incubation at 4°C with primary anti-Ki67 antibody (28074-1-AP, Proteintech) overnight at 4°C, followed by HRP-conjugated secondary antibody and DAB development. For Tunnel immunofluorescence, deparaffinized sections were permeabilized with 0.1% Triton X-100, then incubated with TUNEL reaction mixture (C1088, Beyotime) for 1h at 37°C in dark, counterstained with DAPI.

*Immunology-related Flow Cytometry Analysis:* Freshly harvested spleen and tumor tissues were enzymatically digested using collagenase IV and DNase I at 37°C for 30 min, sequentially filtered through 200-μm nylon mesh, subjected to RBC lysis, and washed twice with ice-cold PBS before incubation with fluorochrome-conjugated antibodies (30 min, 4°C). Antibody-stained cells were fixed with 4% paraformaldehyde and analyzed on a CytoFlex flow cytometer (Beckman Coulter). The antibodies used in this study were as follows: CD11b-Pacific Blue (AC0897, Beyotime), CD80-FITC (AC1147, Beyotime), CD206-APC (A25660, Abclonal), CD3-APC (AC0938, Beyotime), CD4-FITC (AC1104, Beyotime), and CD8-PE (AC1262, Beyotime).

*Elisa:* Freshly dissected murine tumor tissues were homogenized and analyzed using ELISA kits (AiFang Biological) according to manufacturer protocols: IFN-γ (AF2182-A), TNF-α (AF2132-A), and IFN-β (AF2662-A). Briefly, standards and samples were loaded in triplicate onto pre-coated plates alongside blank controls, followed by 37°C incubation (30 min), five washes, HRP-conjugate addition (excluded in blanks), repeated incubation/washing, 10-minute chromogenic reaction (substrates A/B, 37°C protected from light), and termination. Optical density was measured at 450 nm within 15 min post-termination using a microplate reader, with blank-adjusted values used for calculations.

*Blood Biochemistry:* liver/kidney six-item panel blood biochemistry was performed by collecting retro-orbital venous blood samples from mice into serum separator tube, followed by centrifugation at 3,000 rpm for 15 min at 4°C to separate plasma. Samples were transported on dry ice to Soochow University Institute of Health and Environmental Technology Co., Ltd. (Suzhou, China) for automated analysis using a Hitachi 7080 clinical analyzer.

*Complete Blood Count:* CBC analysis was performed by collecting retro-orbital blood samples into EDTA-anticoagulated tubes, gently inverted 8-10 times for mixing. Samples were analyzed within 2 hours at room temperature using a SYSMEX XN-10[B1] automated hematology analyzer (Kobe, Japan).

**Table S1.** Targeted editing site sequences of POLD4 in five distinct CRISPR-Cas9 sgRNA plasmids

| Name | Target sequences (5’-3’) |
| --- | --- |
| Control | ACGGAGGCTAAGCGTCGCAA |
| sgRNA1 | CCTGGGTGGCCGCCATGGGT |
| sgRNA2 | GGGCCCCCTGGGCACAGCAA |
| sgRNA3 | TGAGGCAGTTTGACCTGGCC |
| sgRNA4 | CTATCCTGTTGTGAAGAAGA |

**Table S2.** List of primer sequences utilized in this research

| Name | Sequences (5’-3’) |
| --- | --- |
| POLD4-Forward (for sequencing) | TGCTGGATGGCAAAGGCAAA |
| POLD4-Reverse (for sequencing) | TCCTGTGGCTCTGGTGAACA |
| POLD4-Forward | GTACCCCCACGTTGTCCTTC |
| POLD4-Reverse | GAATGAGCAGAGCAGGCCAT |
| RAD51-Forward | TCACGGTTAGAGCAGTGTGG |
| RAD51-Reverse | ACTCAGTTGCCGTGGTGAAA |
| Bax-Forward | AGGGGCCTTTTTGCTACAGG |
| Bax-Reverse | CACTCGCTCAGCTTCTTGGT |
| Bcl-2-Forward | CCTGTGGATGACTGAGTACCTG |
| Bcl-2-Reverse | AGCCAGGAGAAATCAAACAGAGG |
| Actin-Forward | CATCCGTAAAGACCTCTATGCCAAC |
| Actin-Reverse | ATGGAGCCACCGATCCACA |
| Off-target 1-Forward | AATTACCCAGGCCTCTAAGTGAC |
| Off-target 1-Reverse | GTAAGATTTGGGGTGGGAGAAC |
| Off-target 2-Forward | CGGTATTTGAGTGCGTGTCCT |
| Off-target 2-Reverse | CTTATAAAACAAGGTCAAGCCGAC |
| Off-target 3-Forward | GGAGAGCTGACTGGGACAAC |
| Off-target 3-Reverse | CTCCCGTACCCCCGTATAGT |
| Off-target 4-Forward | GCTAGAAGACGGGACCACTG |
| Off-target 4-Reverse | GAACCTGAGGTCTGGGACATT |
| Off-target 5-Forward | AGCCAGGTTGGAGGCTATAC |
| Off-target 5-Reverse | GACCCATGATGCCAAGGCTA |
| Off-target 6-Forward | TTCCAGCCCTCAAGTTCTGC |
| Off-target 6-Reverse | CTTCCTAACGCACCTCGGAA |


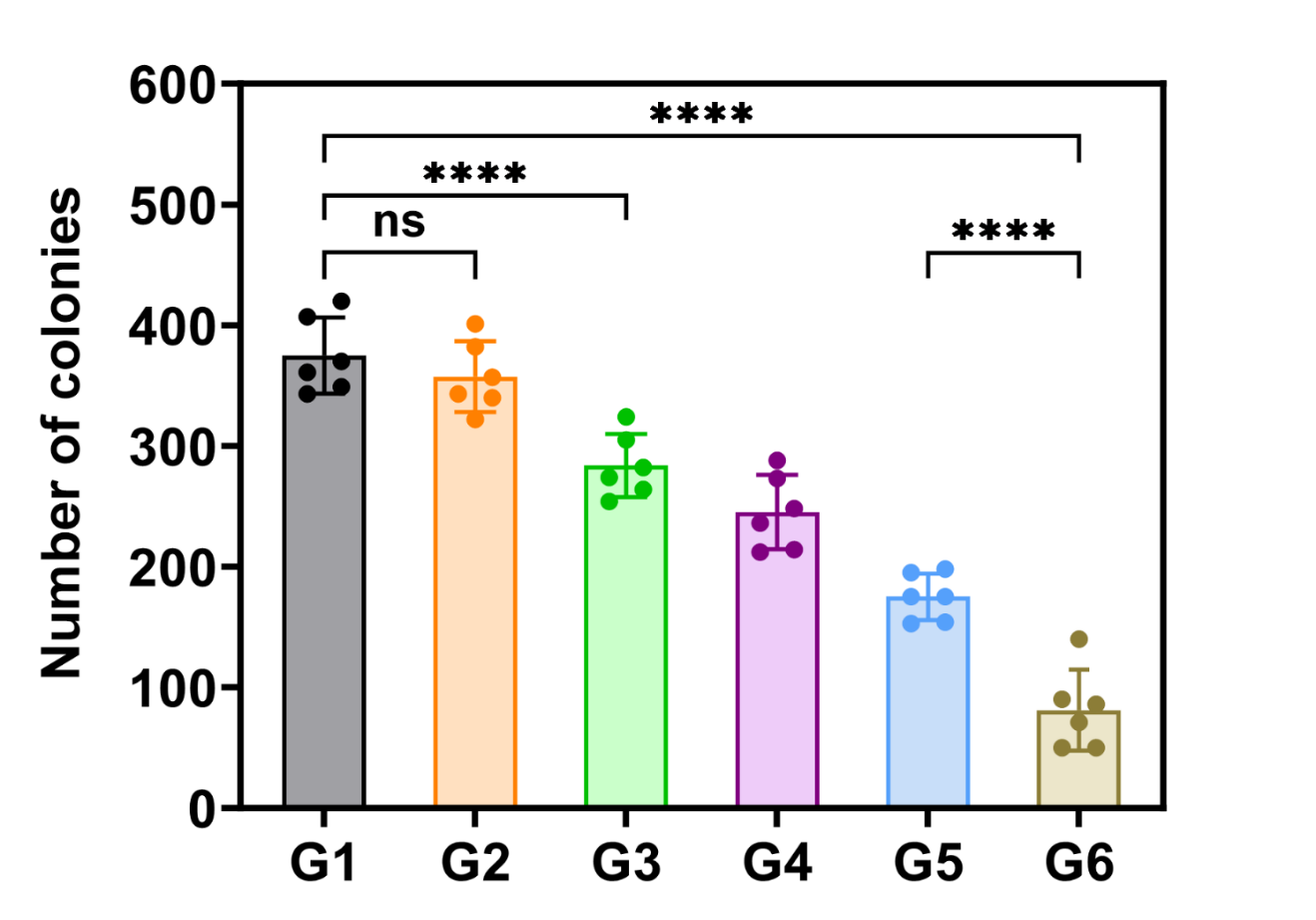


**Figure S1.** Statistical results of colony formation assay in Figure 3B.

**
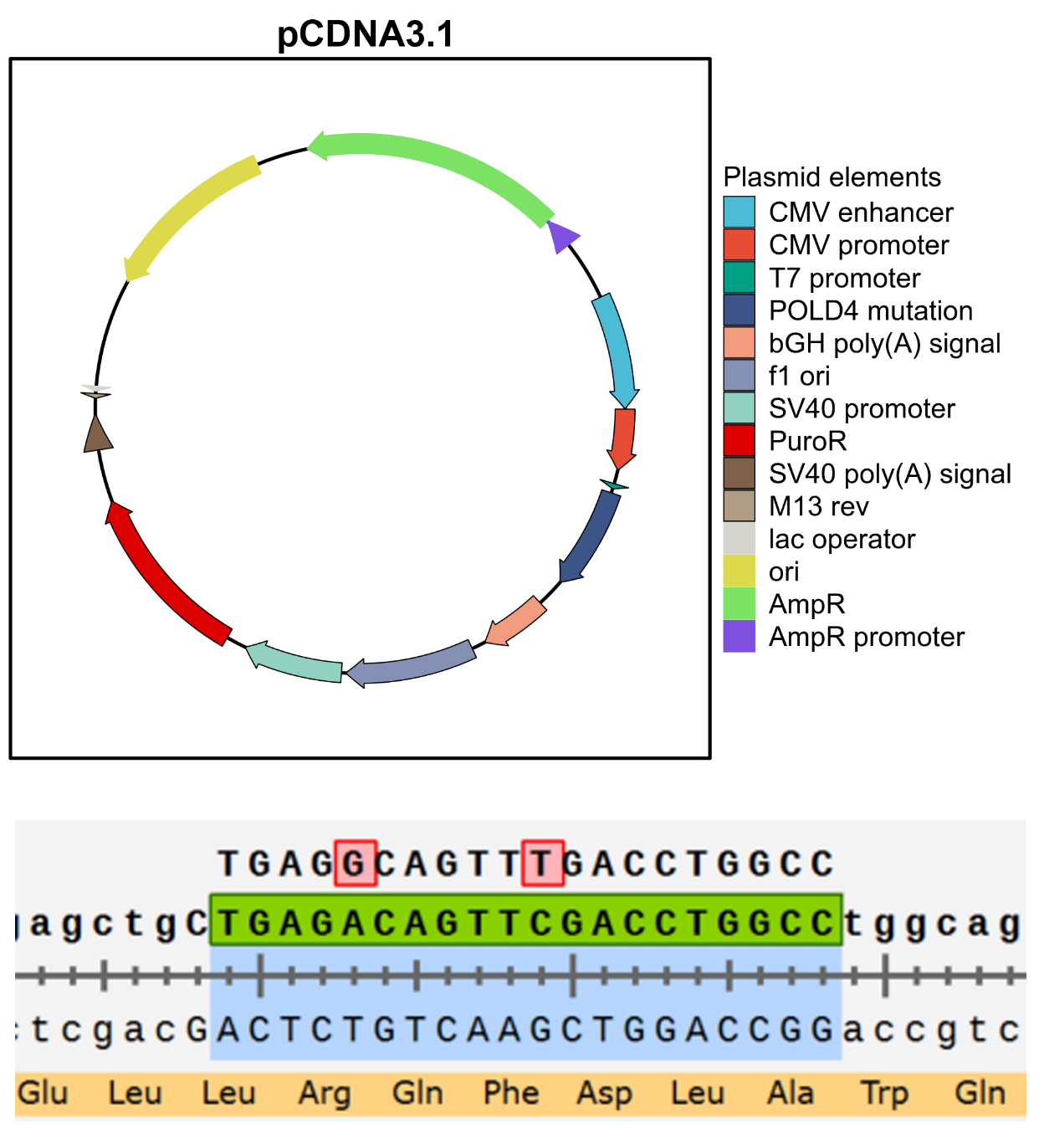
**

**Figure S2.** Construct map of the pcDNA3.1 on-target rescue plasmid (encoding sgRNA-resistant POLD4 cDNA) and target site composition. The target site contains synonymous mutations at two positions, which do not alter POLD4’s amino acid sequence.

**
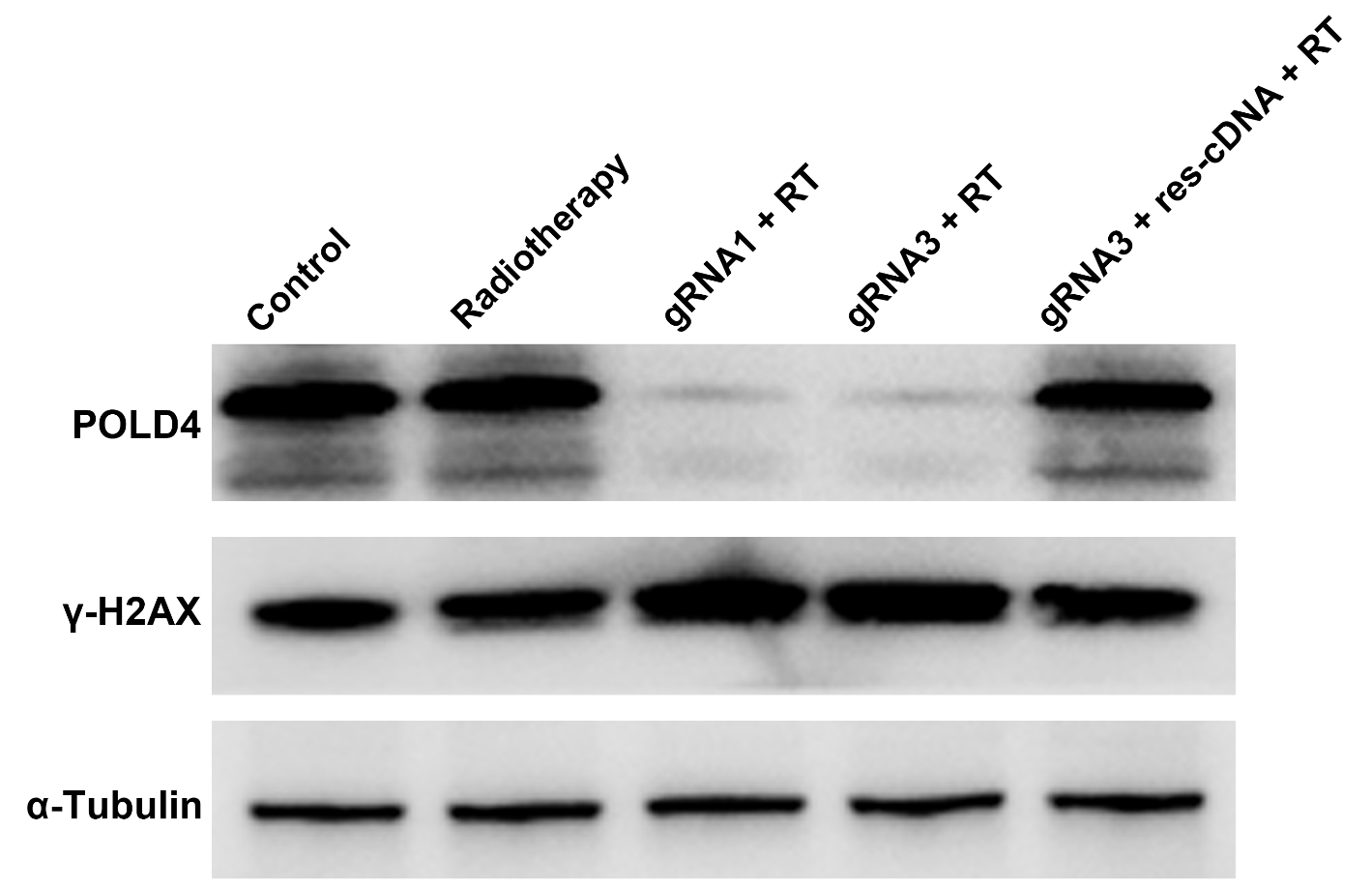
**

**Figure S3.** Western blot analysis of POLD4 and γ-H2AX protein expression in cells treated with radiotherapy, gRNA (gRNA1/gRNA3), and sgRNA-resistant cDNA plasmid. RT, radiotherapy.

**
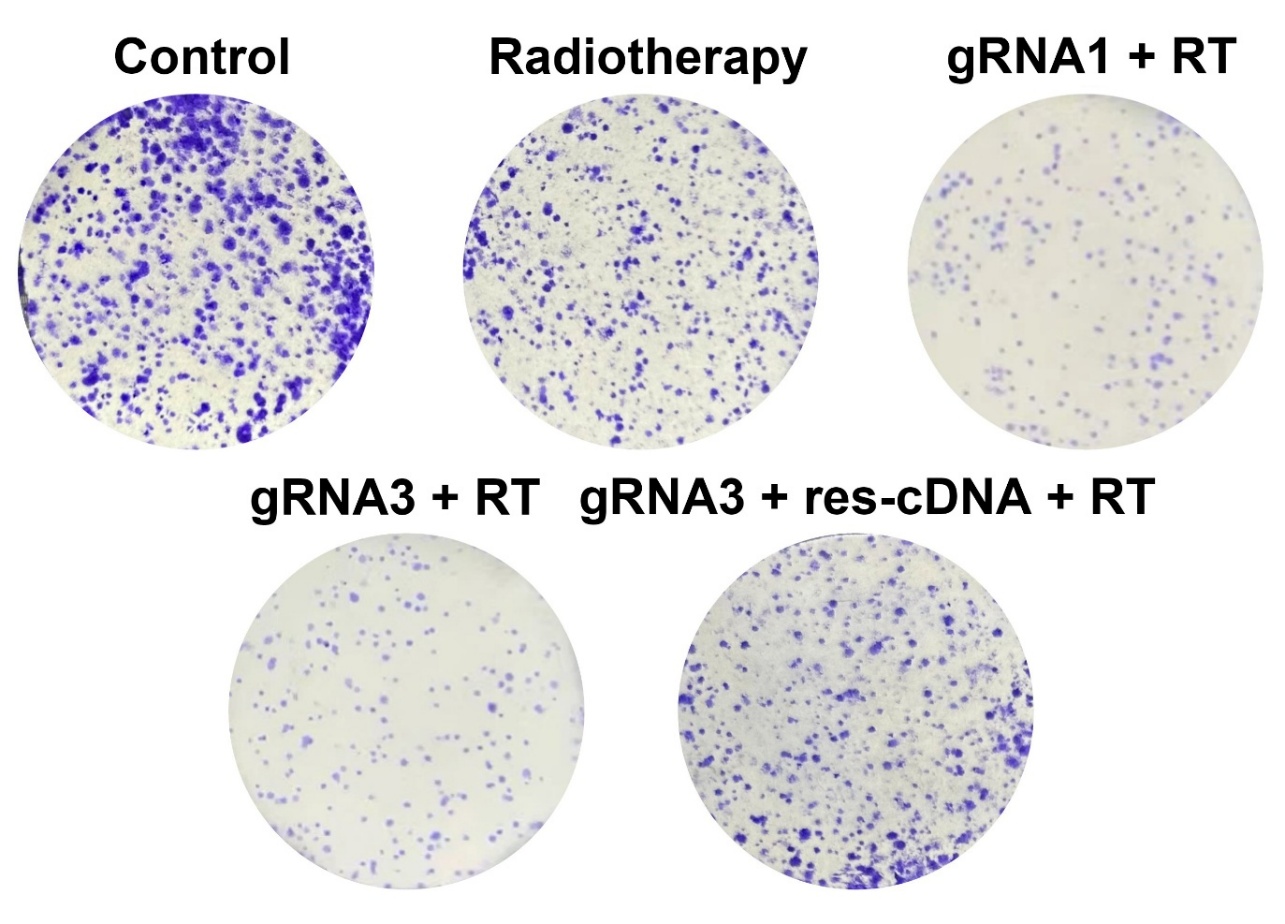
**

**Figure S4.** Colony formation assay results of cells treated with radiotherapy, gRNA (gRNA1/gRNA3), and sgRNA-resistant cDNA plasmid.

**
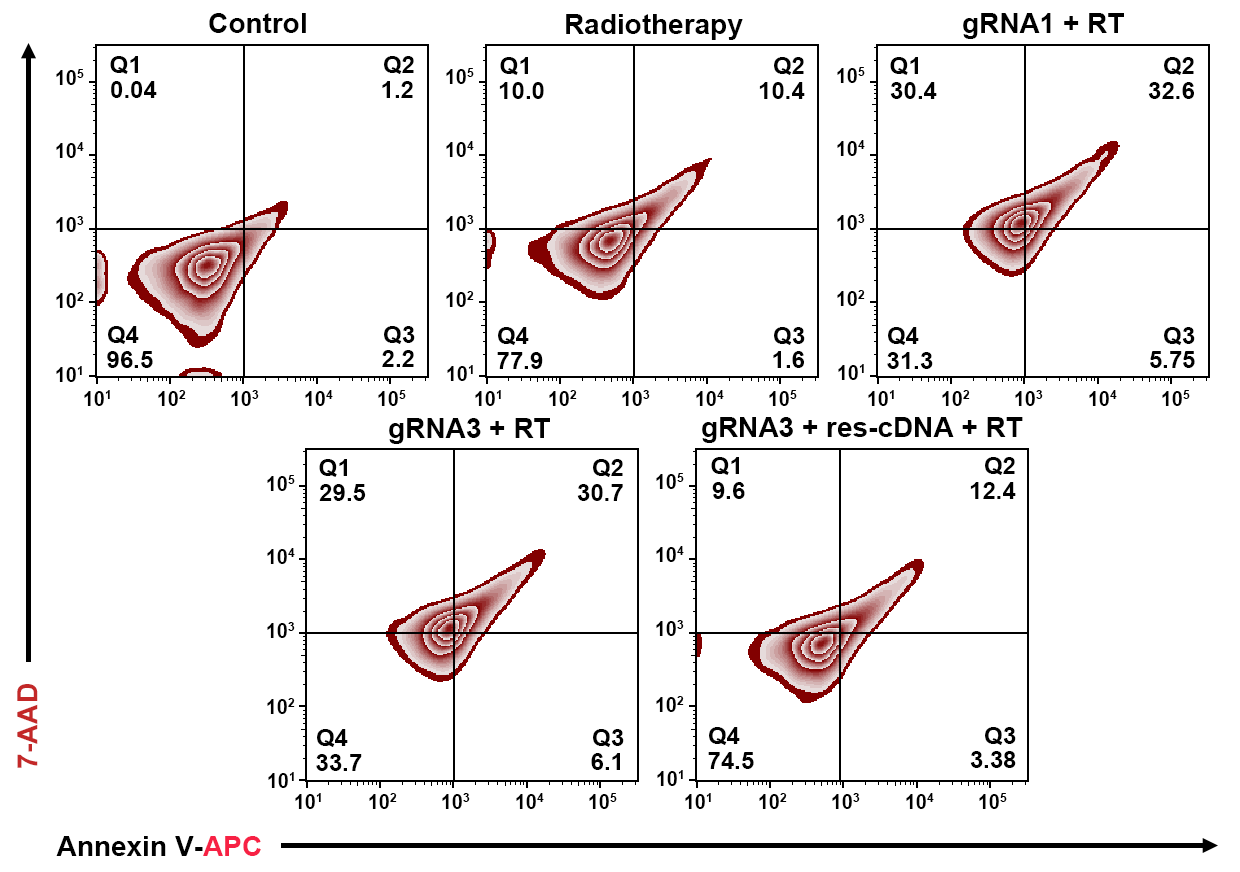
**

**Figure S5.** Flow cytometry analysis of cell apoptosis in cells treated with radiotherapy, gRNA (gRNA1/gRNA3), and sgRNA-resistant cDNA plasmid.

**
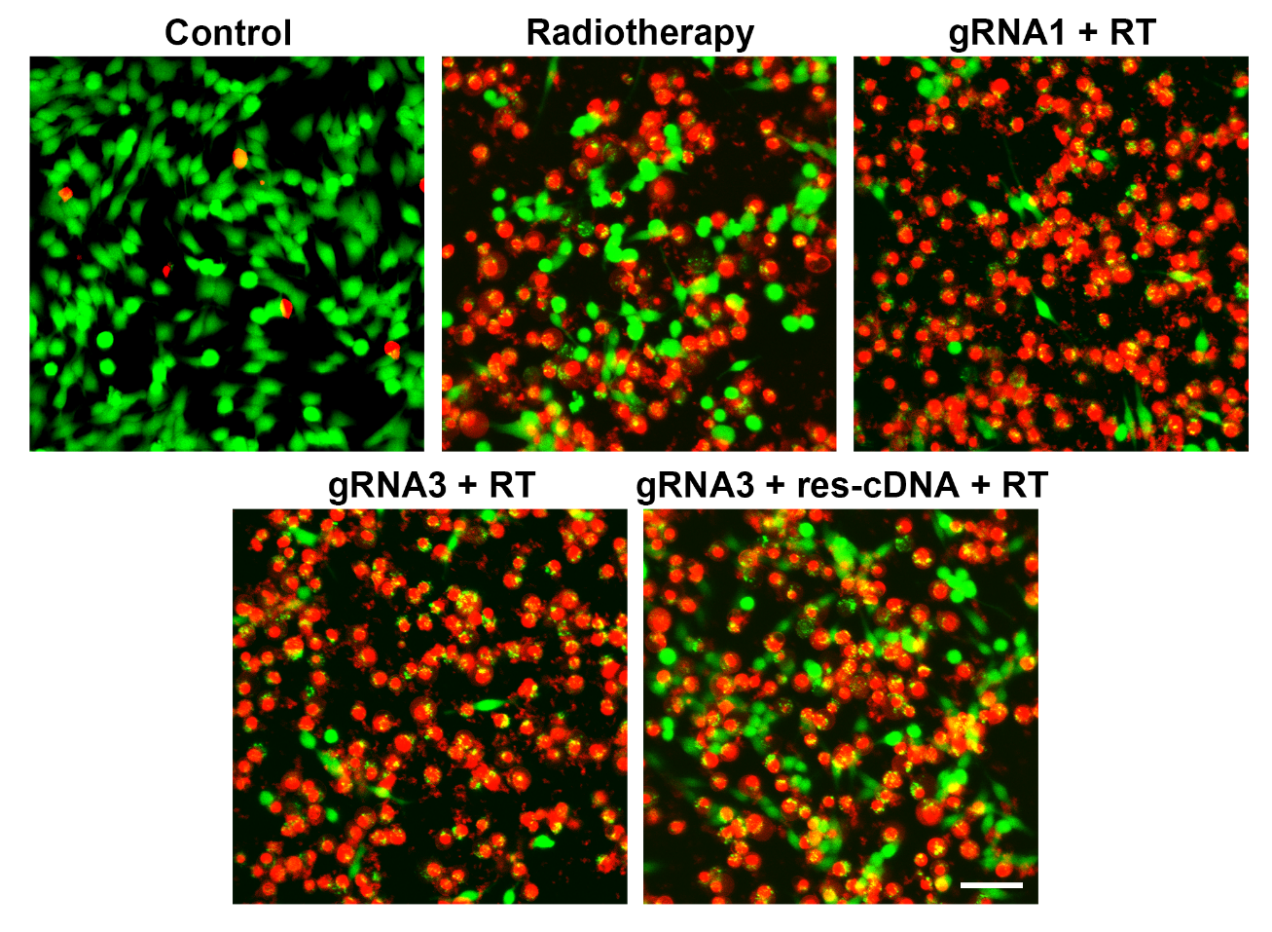
**

**Figure S6.** Live/dead cell staining assay of cells treated with radiotherapy, gRNA (gRNA1/gRNA3), and sgRNA-resistant cDNA plasmid (scale bar: 50 μm).

**
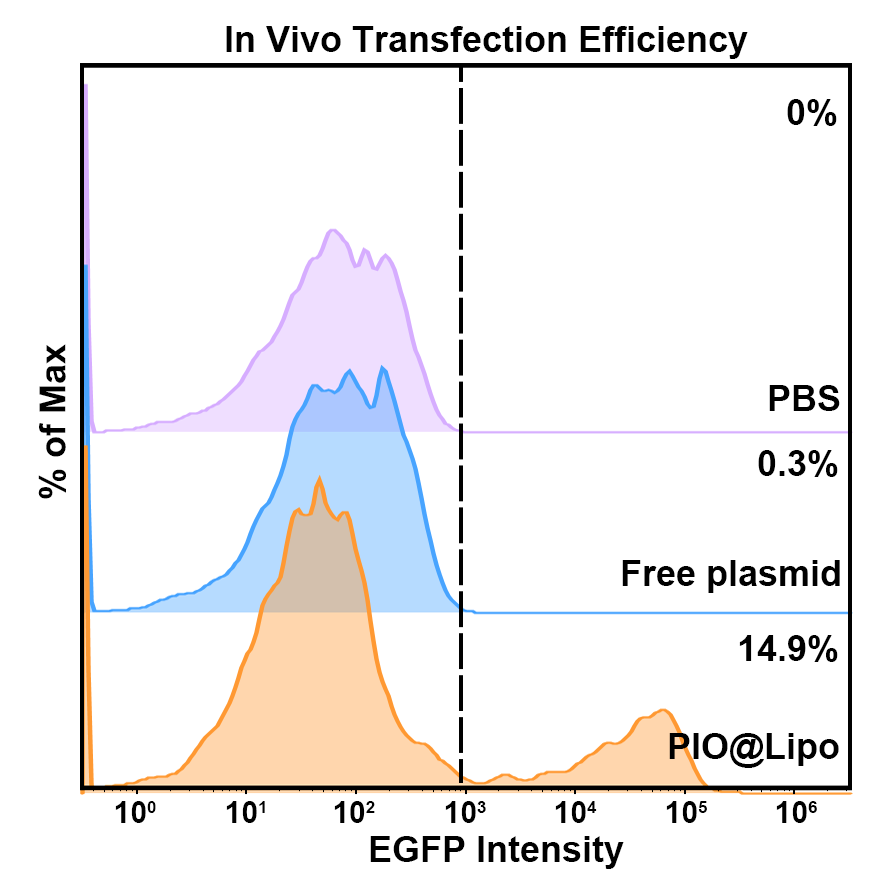
**

**Figure S7.** In vivo transfection efficiency assessment of PIO@Lipo.

**
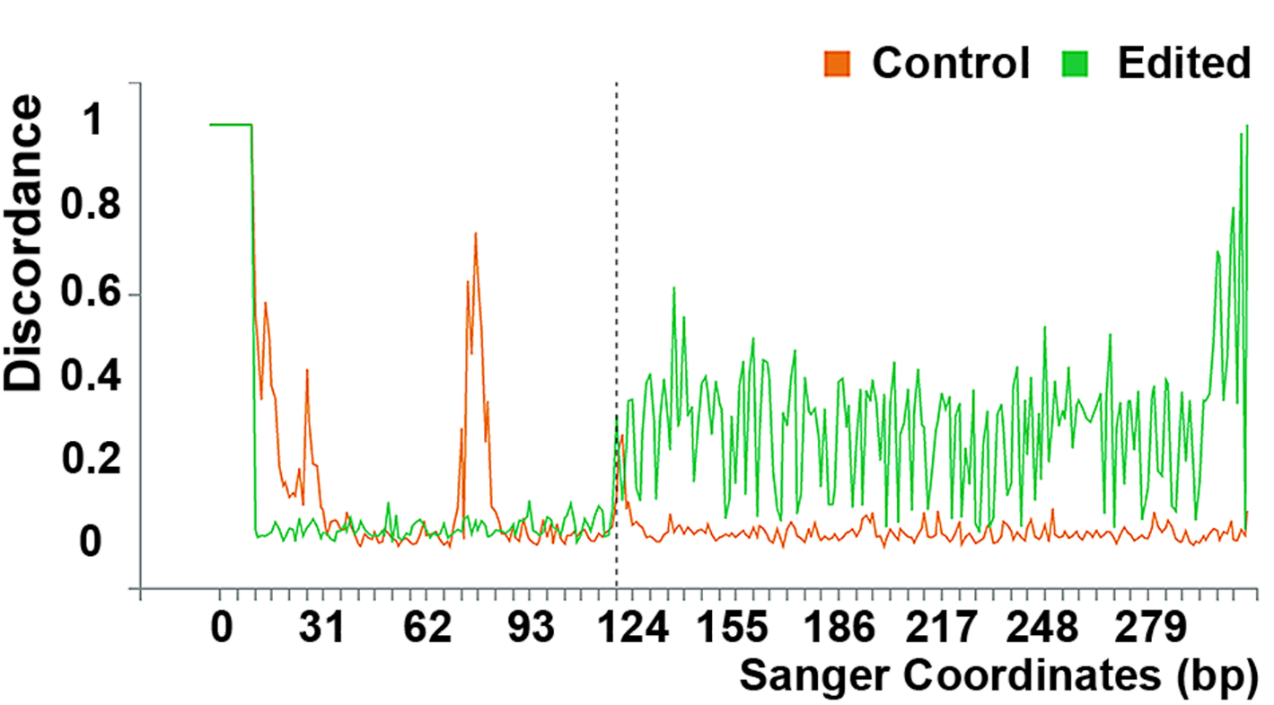
**

**Figure S8.** Sanger sequencing analysis of the POLD4 target site in PIO@Lipo-treated cells. It shows significant genetic discordance between the gene-edited (Edited) group and the Control group.

**
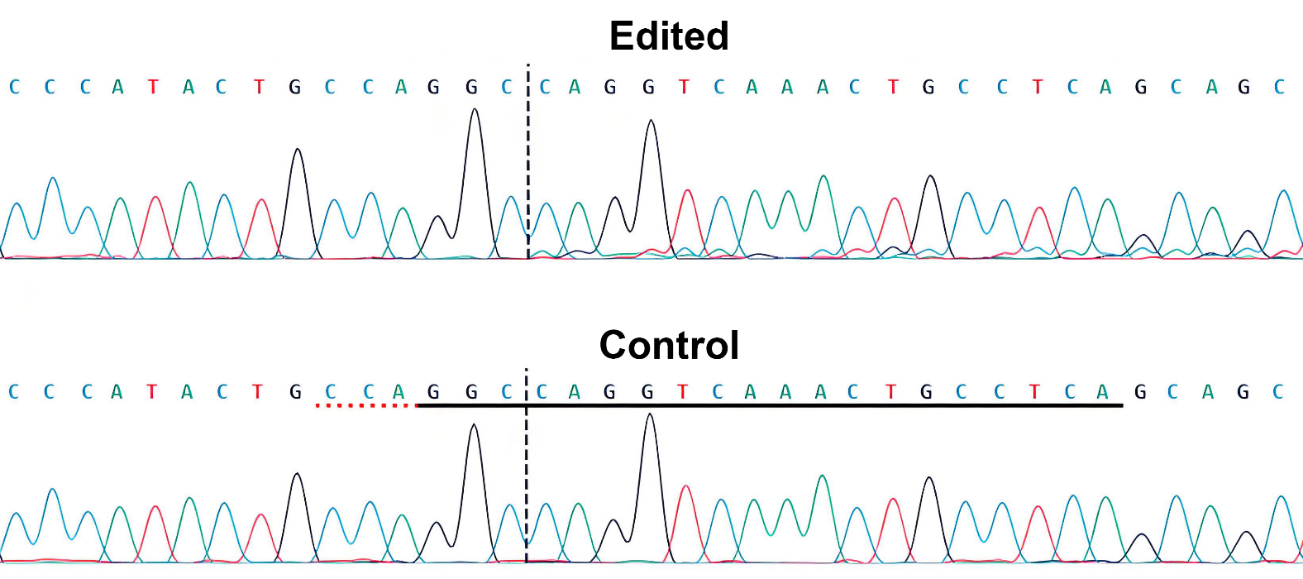
**

**Figure S9.** In vivo Sanger sequencing profiles of POLD4 in PIO@Lipo-treated animal samples.

**
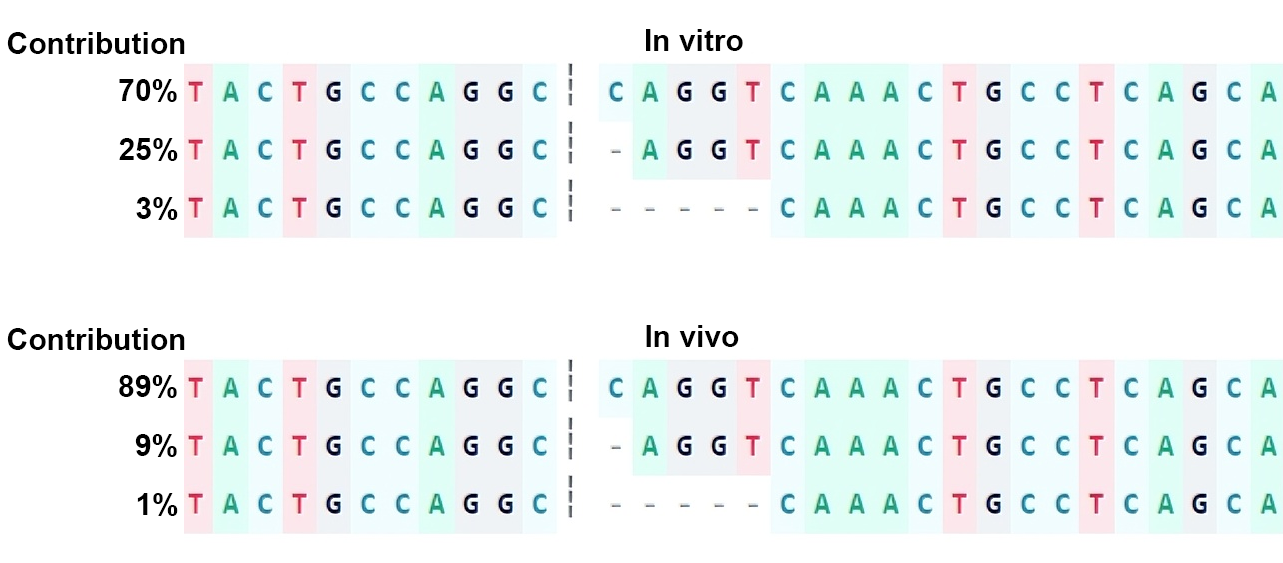
**

**Figure S10.** ICE analysis of Indel frequencies in the POLD4 gene from in vitro and in vivo PIO@Lipo-treated samples. The numbers on the left represent Indel%, and the dashed lines indicate the CRISPR cleavage sites.

**Table S3.** Information for the six off-target sites

| Name | Target sequences (5’-3’) | PAM | Locus |
| --- | --- | --- | --- |
| On-target | TGAGGCAGTTTGACCTGGCC | TGG | chr19:4282565 |
| Off-target 1 | TGAGCCAGTTTGACAAGGCC | TGG | chr1:19936452 |
| Off-target 2 | TGATGCAGCTTGACCTGGCC | TGG | chr7:132237746 |
| Off-target 3 | TGAGGCAGTCTGAGCTAGCC | AGG | chr15:78716252 |
| Off-target 4 | TGTGGCAGTTTGAGCTGTCC | AGG | chr7:109671564 |
| Off-target 5 | ACAGGTAGTTTGACCTGGCC | TGG | chr11:93452904 |
| Off-target 6 | TGGGGCAGTTGCACCTGGCC | TGG | chr8:70564747 |

PAM, Protospacer Adjacent Motif.

**
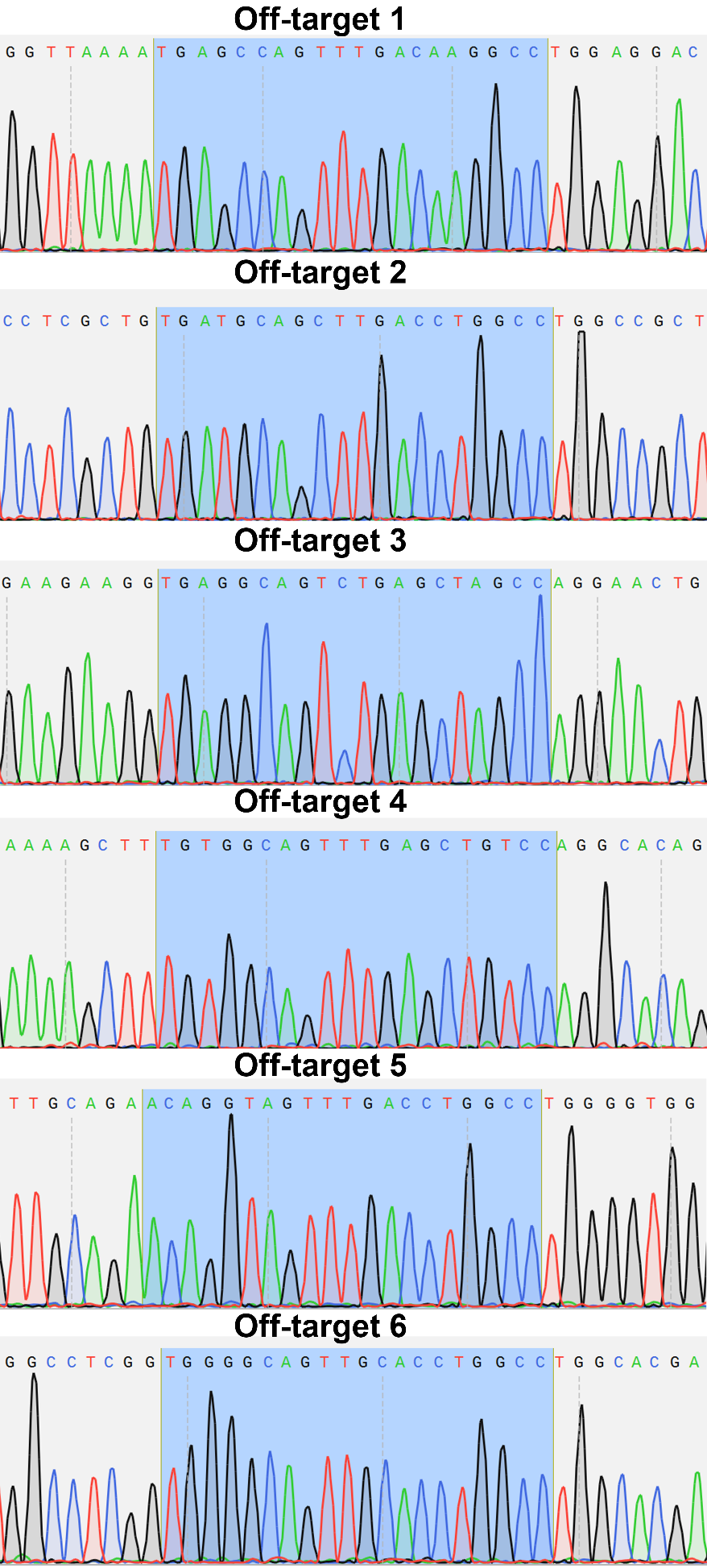
**

**Figure S11.**Sanger sequencing results of the six off-target sites.

**
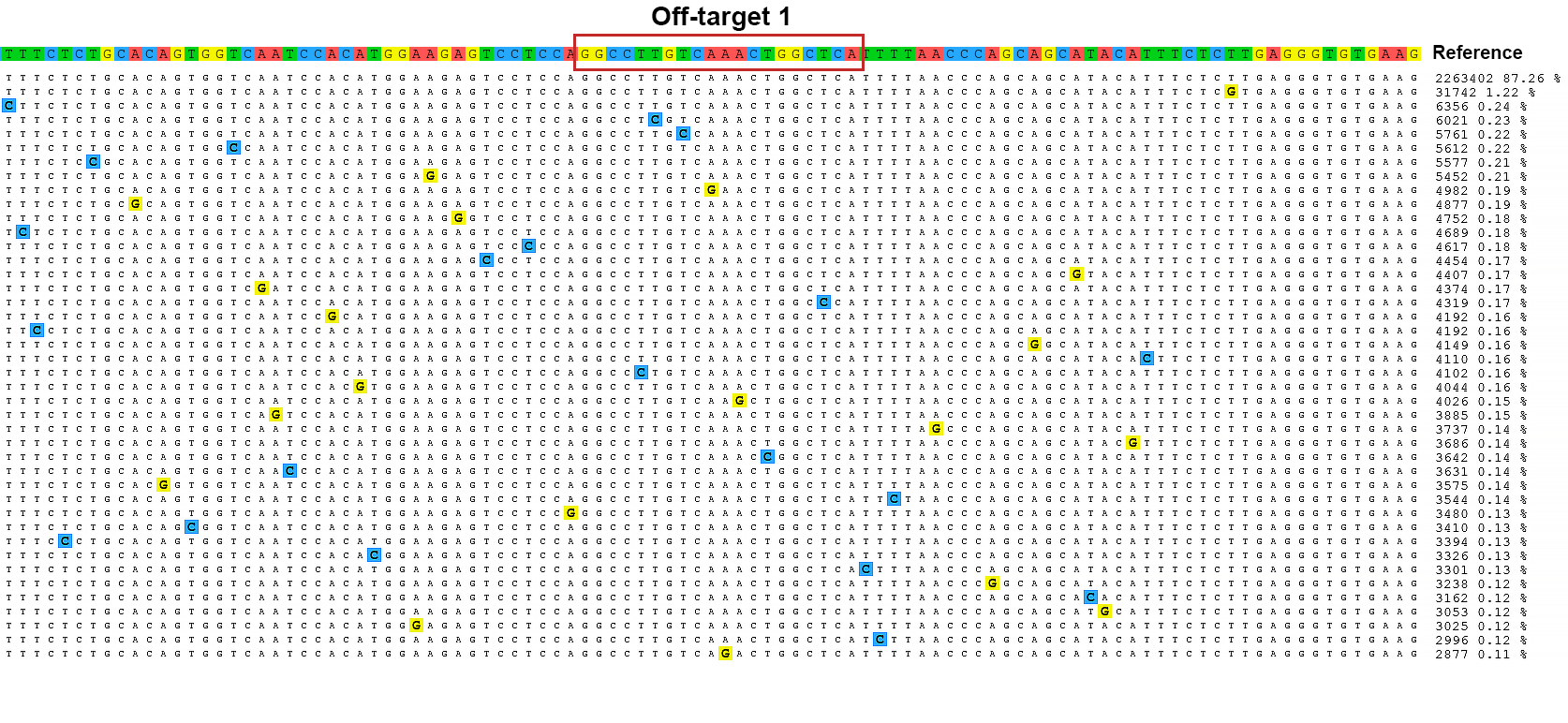
**

**Figure S12.** Sequence alignment of the amplicon sequencing region for off-target 1.

**
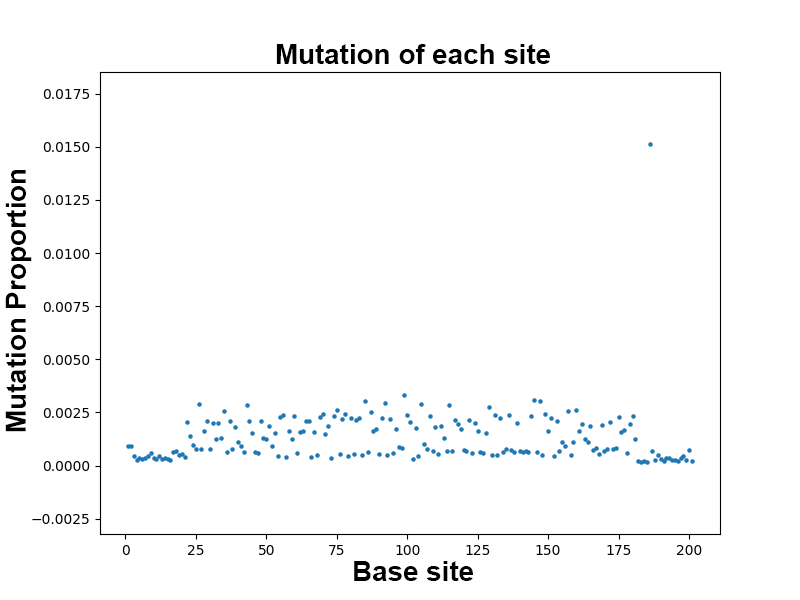
**

**Figure S13.** Mutation proportion of each base site in the amplicon sequencing region of off-target 1.


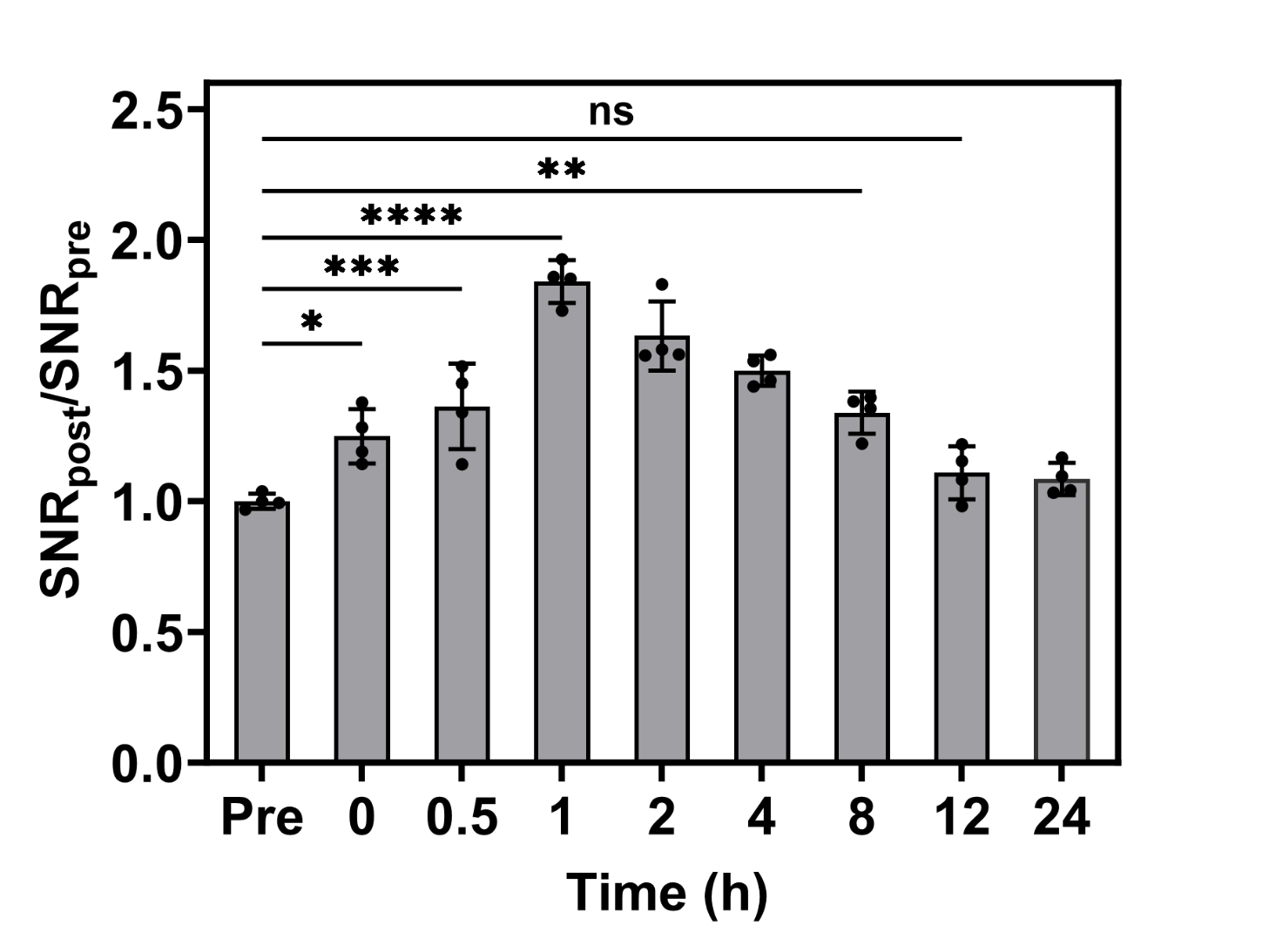


**Figure S14.** Signal-to-noise ratio statistical analysis of PIO@Lipo in animal MRI.


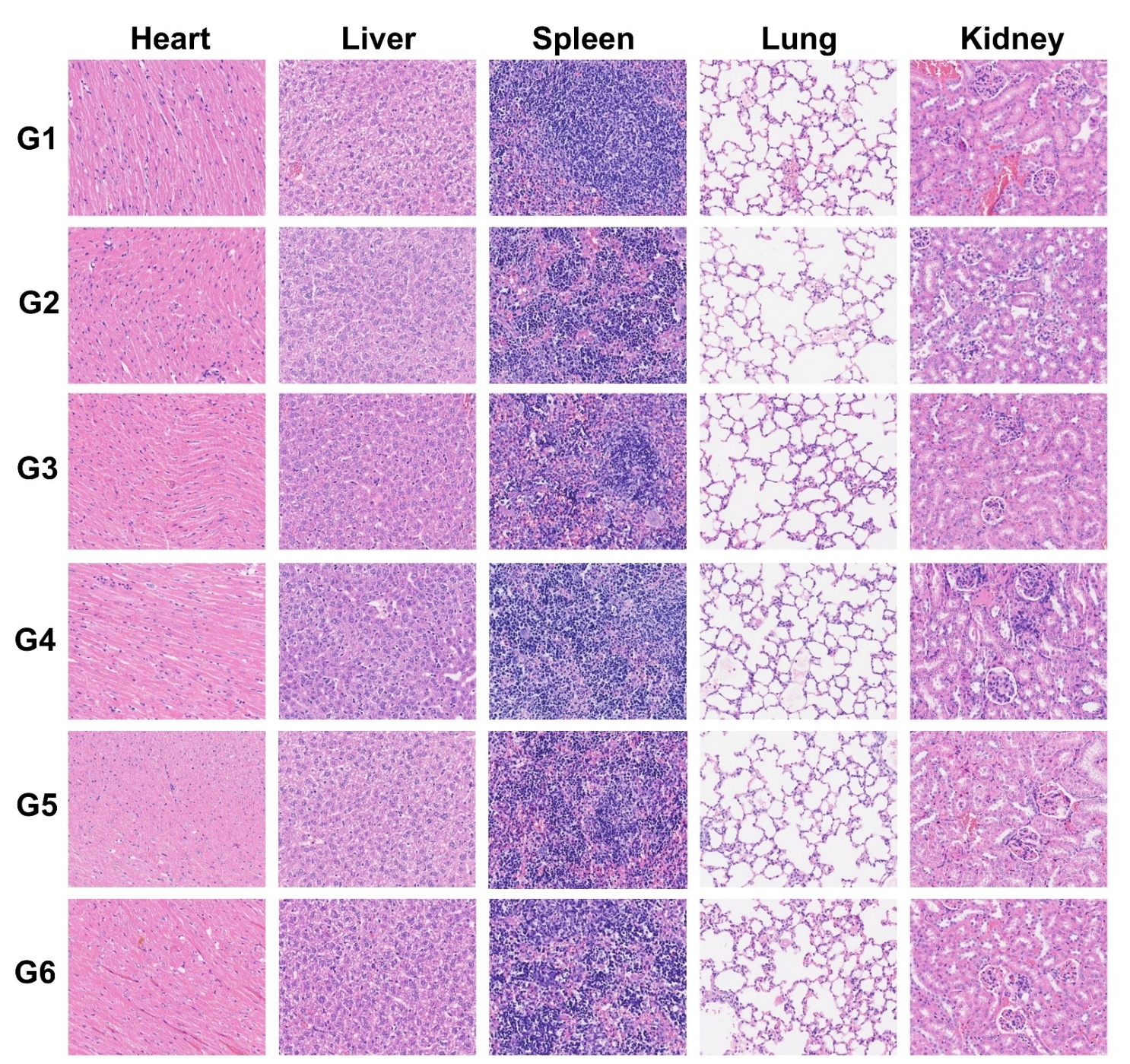


**Figure S15.** H&E staining of key organs in C57BL/6J mice


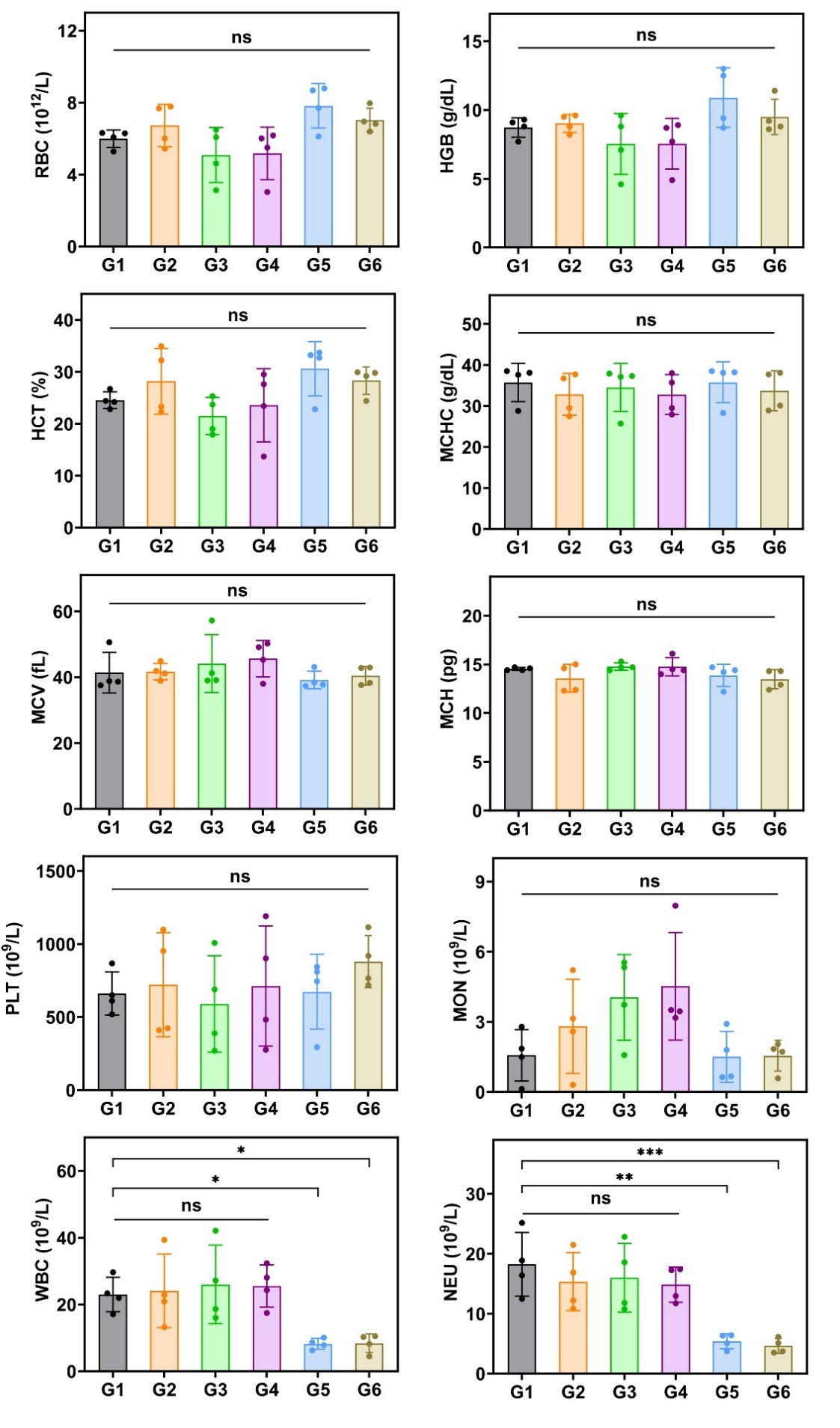


**Figure S16.** Complete blood count analysis in C57BL/6J mice following treatment.
